# Unifying the hallmarks of major depression through neuroimmune–metabolic–oxidative (NIMETOX) dysregulation: a mechanistic systems framework

**DOI:** 10.1038/s41423-026-01448-1

**Published:** 2026-07-13

**Authors:** Michael Maes, Abbas F. Almulla, Drozdstoj Stoyanov, Yingqiang Zhang

**Affiliations:** 1 Sichuan Provincial Center for Mental Health, Sichuan Provincial People’s Hospital, School of Medicine, University of Electronic Science and Technology of China, Chengdu, China; 2 Key Laboratory of Psychosomatic Medicine, Chinese Academy of Medical Sciences, Chengdu, China; 3 Department of Psychiatry, Faculty of Medicine, The Thai Red Cross Society, Chulalongkorn University, and King Chulalongkorn Memorial Hospital, Bangkok, Thailand; 4 Department of Psychiatry, Medical University of Plovdiv, Plovdiv, Bulgaria; 5 Research Center, Medical University of Plovdiv, Plovdiv, Bulgaria; 6 Research and Innovation Program for the Development of MUs - PLOVDIV– (SRIPD-MUP), Creation of a network of research higher schools, National plan for recovery and sustainability, European Union – NextGenerationEU, Plovdiv, Bulgaria; 7 Kyung Hee University, Seoul, Korea; 8 Medical Laboratory Technology Department, College of Medical Technology, The Islamic University, Najaf, Iraq

**Keywords:** Major depression, Inflammation, Atherogenicity, Lipid peroxidation, Reverse cholesterol transport, Diagnostic markers, Neuroimmunology

## Abstract

Current state-of-the-art neuroimmune, metabolic, and oxidative stress (NIMETOX) knowledge that has been developed in clinical major depressive disorder (MDD) research over the past three decades is explored in this review. Between 1990 and 2000, the acute phase of severe MDD was characterized by the activation of T helper (Th)1 cells and M1 macrophages, leading to immune dysregulation that affects nutritional immunity and alters protein, tryptophan, iron, and lipid metabolism. The latter comprises lower high-density lipoprotein cholesterol, reverse cholesterol transport (RCT), ω3 polyunsaturated fatty acids, heightened lipid peroxidation and atherogenicity. Additionally, immune alterations regulate stress-responsive systems and modify the biological basis of depressive symptoms through neurotoxic effects and reduced neuroprotection. The incremental information acquired from 2000 to 2026 revealed that the acute phase of severe MDD is characterized by immune sensitization, imbalances between the compensatory immunoregulatory system (CIRS) and the immune-inflammatory response system (IRS) and that there are multiple interactions between increased atherogenicity, metabolic syndrome, oxidative stress, and lower antioxidant activity and RCT. Additionally, the NIMETOX pathway may be fuelled by increased expression of TLR4 and NF-κB intracellular signaling driven by increased lipopolysaccharides, lipids, and oxidatively modified epitopes. This paper presents evidence that peripheral NIMETOX pathways may lead to neuroinflammation, microglial activation, and neuronal damage and that increased lipid load impairs these central pathways. This paper assesses the field’s future advancements by conducting a comprehensive examination of the reviewed knowledge base, deep phenotyping, panomics methodologies, and machine learning techniques, including the nomothetic precision approach.

## Introduction

By 2026, research has indicated that major depressive disorder (MDD) is associated with abnormalities in neuroimmune, metabolic, and oxidative stress (NIMETOX), which seem to influence its pathophysiology and causation [1]. Immune disorders during the acute phase of severe MDD are intricate and involve the activation of the immune–inflammatory response system (IRS), which is characterized by macrophage M1 activation and T helper (Th)1 and Th17 immune profiles, in addition to relative deficiencies in the compensatory immunoregulatory system (CIRS), which includes M2, Th-2, and T regulatory (Treg) cell profiles [2, 3]. Furthermore, the acute phase of severe MDD is characterized by the emergence of activated T cells and a reduced quantity of Treg cells [4]. Major metabolic disorders in MDD include abnormalities in lipid metabolism characterized by diminished reverse cholesterol transport (RCT) and heightened atherogenicity, disruptions in protein metabolism resulting in reduced total serum protein and decreased plasma tryptophan availability to the brain, and nutritional immunity with immune-linked alterations in trace elements such as iron and zinc [5, 6]. Finally, the acute phase of MDD is characterized by heightened oxidative and nitrosative stress (O&NS) together with diminished antioxidant defenses [7].

Furthermore, it has recently become evident that the clinical heterogeneity of MDD obstructs the interpretation and diagnostic utility of NIMETOX (and other) biomarkers [8, 9]. Different depression phenotypes may yield conflicting results, and certain clinical features of MDD influence these pathways [10]. Furthermore, the literature is replete with insufficient concepts, such as “inflammatory depression” or “immune-mediated depression,” and the classification of depression on the basis of these flawed concepts further obfuscates the interpretation and understanding of the NIMETOX pathways in MDD.

To understand the various NIMETOX pathways and their interconnections, we will examine the current state-of-the-art NIMETOX knowledge developed in clinical research during the past three decades. We first examine the principal discoveries from 1990 to 2000, followed by a review of the incremental information acquired from 2000 to 2026, thus summarizing the current state of the art as of 2026. We will evaluate future advancements in the field through a thorough analysis of the existing knowledge base, deep phenotyping, panomics methodologies, and machine learning.

## Discoveries 1990–2000

### Increased production of Th and macrophage cytokines

Prior to 1990, it was believed that MDD was associated with immunosuppression [11]. This was confirmed through the results of lymphocyte proliferation assays (LPA) and natural killer cell activity (NKCA) assays. Moreover, numerous studies have demonstrated that MDD is associated with elevated serum and urinary cortisol levels and the escape of cortisol and ACTH from the suppressive effects of dexamethasone [12, 13]. These results suggest that the hypothalamic–pituitary–adrenal (HPA) axis is significantly hyperactive. Maes et al. [14] reported that the immune system activates the HPA axis, which then feeds back to the immune system in MDD.

Therefore, the laboratories of Dr. Maes initiated research into the mechanisms that could account for the reduced immune function in MDD. Consequently, the primary cytokines that were recognized at that time to be responsible for immune functions, such as interleukin (IL)-1β, IL-6, and IL-2, as well as the soluble IL-2 receptor (sIL-2R), were investigated. Furthermore, the measurement of cytokines was a methodologically challenging endeavor at that time because of the absence of sensitive assays. Consequently, in 1988–1990, the Maes laboratory was able to investigate IL-6 and IL-1β only after stimulating peripheral blood mononuclear cells (PBMCs) with in vitro immune activators.

Our initial hypothesis was that MDD is linked to a reduction in the secretion and production of critical cytokines that influence the immune system response, including IL-1β, IL-6, IL-2, and interferon-gamma (IFN-γ). The results of the immune biomarker discoveries published between 1990 and 2000 are summarized in Table 1.

**Table 1:** Discoveries on the role of the neuroimmune, metabolic, and oxidative stress (NIMETOX) pathways in major depression (MDD) and psychological stress occurred in the 1990s

Biomarkers	Illness	Authors	PubMed ID
**Discoveries IRS activation**			
Increased serum interleukin (IL)-2 and soluble IL-2 receptor (sIL-2R)	MDD	Maes et al. 1990 [15]	2135065
Increased stimulated IL-1β	MDD	Maes et al. 1991 [16]	1746291
Activated HLA-DR + T, CD25 + T, and CD19 + B	MDD	Maes et al. [24]	1574566
Acute phase proteins (APP) and complement C3C	MDD	Maes et al. [34]	1573127
APP response in melancholia	MDD	Maes et al. [35]	1379370
B-cell proliferation (CD20 + , CD21 + )	MDD	Maes et al. [25]	1573121
T-cell activation (CD7 + CD25 + , CD2 + HLA-DR + )	MDD	Maes et al. [26]	8430217
Increased stimulated IL-6	MDD	Maes et al. [17]	7511248
Increased stimulated interferon-γ	MDD	Maes et al. [18]	7761549
Immune activation causes immunosuppression	MDD	Maes et al. [30]	7938562
Increased serum IL-6, sIL-6R, sIL-2R and s transferrin receptor (sTfR)	MDE/UP/BD	Maes et al. [19]	8550956
Increased serum sIL-6R and sIL-2R	BD/mania	Maes et al. [42]	7666381
Increased serum sIL-1R antagonist (sIL-1RA)	MDD	Maes et al. [31]	9367546
Increased serum C-reactive protein in association with IL-6 and sIL-6R	MDD	Sluzewska et al. [21]	8944394
APP response in mania and bipolar (BP) disorder	BD/mania	Maes et al. [43]	9061799
Lowered natural immunoregulation	MDD	Maes et al. [32]	10579545
Increased serum tumor necrosis factor (TNF)-α	MDD	Mikova et al. 2001 [22]	11418279
**Discoveries of immune-lipid metabolism interactions**			
IgG against anticardiolipin antibodies	MDD	Maes et al., [54]	1851504
IgG against antiphosphatidylserine and antipartial thromboplastin antibodies	MDD	Maes et al.[55]	8465663
Lower lecithin cholesterol acyl transferase (LCAT) activity and reverse cholesterol transport	MDD	Maes et al. [52]	7831994
Lower ω3 polyunsaturated fatty acids in phospholipids	MDD	Maes et al. [57]	8735157
Lower high-density lipoprotein, increased atherogenicity and immune activation	MDD	Maes et al. [51]	9111854
Lower ω3 PUFAs due to oxidative stress and lower antioxidant defenses (serum zinc)	MDD	Maes et al. [58]	10333380
Lipid – Cytokine – interactions	MDD	Maes and Smith [61]	9513744
Lower vitamin E + lower antioxidant defenses	MDD	Maes et al. [62]	10802134
**Discoveries of immune-protein metabolism interactions**			
Higher positive APPs, lower negative APPs	MDD	Maes [44]	7506108
Lower tryptophan is an indicator of immune activation	MDD	Maes et al. [242]	7908745
Lower tryptophan may be caused by lower albumin and higher indoleamine-2,3-dioxygenase activation in melancholia	MDD	Maes et al.[18]	7761549
Lowered total serum protein and albumin and immune activation	MDD	Van Hunsel et al. [66]	9029664
**Discoveries nutritional immunity**			
Lower iron, erythron aberrations and IRS activation	MDD	Maes et al. [5]	8882911
Lower zinc and IRS activation	MDD	Maes et al. [6]	9276075
**Discoveries IRS activation - HPA-axis interactions**			
Increased glucocorticoid suppression of sIL-2R and IL-1β	MDD	Maes et al. [16]	1746291
Associations between IL-1β and hypothalamic‒pituitary‒adrenal (HPA) axis hyperactivity	MDD	Maes et al. [79]	8328562
Associations between IL-6 and HPA-axis hyperactivity	MDD	Maes et al. [17]	7511248
Associations IL-6, sIL-2R, and serum cortisol	MDD	Maes et al. [80]	7669825
**Discoveries effects psychological stress on IRS**			
M1, T helper 1 and Treg activation, T helper 1 polarization	Stress	Maes et al. [93]	3617578
T-cell activation (CD2 + CD69+ and CD2 + HLA-DR + T cells), and B-cell proliferation (CD19 + )	Stress	Maes et al. [92]	9892853
Lowered immunoregulation (CC16 and sgp130)	Stress	Song et al. [94]	10333381
Lower Albumin, changes in protein metabolism	Stress	Van Hunsel et al. [243]	9572088
**Discoveries treatment-resistant depression (TRD)**			
Increased IL-6 and sIL-1RA	TRD	Maes et al. [31]	9367546
Lowered L-tryptophan	TRD	Maes et al. [68]	9224908
Lowered zinc	TRD	Maes et al. [6]	9276075

The results did not correspond with the a priori hypothesis of immunosuppression in MDD. In contrast, Maes et al. reported that severe MDD in hospitalized patients with no other concomitant medical condition is characterized by IRS activation, specifically T-cell activation with elevated serum levels of sIL-2R, an increased prevalence of measurable IL-2, increased in vitro-stimulated production of sIL-2R and interferon (IFN)-γ, and macrophage activation, including increased IL-1β and IL-6 production [15–18].

In an American study sample, patients with MDD and healthy controls exhibited elevated IL-6 and sIL-6R levels, as well as elevated sIL-2R levels [19]. It was previously recognized that sIL-6R could bind to IL-6 and that this complex could enhance the signaling of IL-6, a process that was subsequently referred to as IL-6 trans-signaling [20]. The findings of a simultaneous increase in IL-6 and sIL-6R were replicated in a Polish study sample [21]. Increased serum levels of other proinflammatory cytokines, namely, tumor necrosis factor (TNF)-α, were detected in a Bulgarian sample of MDD patients [22]. In Germany, Seidel et al. [23] reported increased production of IL-6, IL-2, IFN-γ, and IL-10 by whole blood.

### T-cell activation

Subsequent to these preliminary discoveries, the Maes laboratories, which were situated at two distinct inpatient clinics in Belgium, intended to investigate additional biomarkers of IRS activation, including the evaluation of acute-phase proteins (APPs) and T-cell activation markers through flow cytometry in severe inpatient MDD (IMDD). Signs of immune cell activation, including increased numbers of white blood cells, monocytes, memory T cells (CD4 + CD45RA + ), activated T cells (e.g., CD7 + CD25+ and CD2 + HLADR + ), and B cells (CD19 + , CD20 + , and CD21 + ), coupled with an increased CD4 + /CD8+ ratio [24–27], were detected in various study populations of IMDD patients. Moreover, T-cell activation markers demonstrated high accuracy for IMDD, with a sensitivity of 64% and a specificity of 91% [26]. When supervised machine learning techniques are employed, Maes et al. [28] demonstrated that controls and IMDD are two qualitatively distinct groups in terms of T-cell activation.

Furthermore, IMDD is accompanied by the activation of cell-mediated immunity (CMI), as evidenced by increased PBMC production of interferon (IFN)-γ and a corresponding increase in serum neopterin levels [18]. The latter is a critical indicator of CMI that is generated by macrophages in response to T-cell markers, including IFN-γ [18]. Neopterin/creatinine ratios are substantially elevated in the plasma of patients with depression [29].

### Immunosuppression and the immunoregulatory system

On the basis of the above results, Maes et al. devised a novel theory that MDD is characterized by concurrent immunosuppression and an IRS response [27]. For instance, the variance in the NKCA can be explained by the number or percentage of leukocytes, monocytes, neutrophils, and activated T cells [30]. Notably, in the 1990s, MDD in inpatients was associated with elevated levels of not only sIL-2R but also sIL-1R antagonist (sIL-1RA) [15, 31]. Notably, both molecules have the potential to reduce IRS responses, specifically IL-2 and IL-1 signaling, although increases in these markers suggest the presence of an IRS response.

In addition, severe IMDD is associated with a reduction in the levels of natural immunoregulatory substances, including Clara cell protein (CC16), which function as inhibitors of IRS activation [32]. Maes et al. [33] suggested that the presence of MDD may be associated with a predisposition to IRS responses due to reduced levels of endogenous immunoregulatory defenses. This is significant because three decades later, it was demonstrated that all different MDD phenotypes are accompanied by imbalances between immunoregulatory CIRS forces and IRS functions, as discussed later.

### The acute phase or mild inflammatory response

In addition, we anticipated detecting elevated levels of positive APPs and decreased levels of negative APPs in the event that severe IMDD is indeed accompanied by IRS activation. In accordance with this theory, compared with healthy controls, IMDD patients exhibit elevated plasma concentrations of haptoglobin, alpha 1 antitrypsin, and ceruloplasmin (positive APPs) and lower levels of negative APPs such as albumin, transferrin, and retinol binding protein [34–36]. In New Zealand, Joyce et al. [37] reported that compared with control men, depressed men had significantly elevated levels of APPs, including haptoglobin, alpha-1-antichymotrypsin, and immunoglobulin G. Similarly, Song et al. [38] reported increased levels of haptoglobin, alpha 1 antitrypsin, immunoglobulin (Ig)M, and the complement factors C3c and C4 in unipolar depressed patients compared with those in healthy controls. Seidel et al. [23] reported increased APPs, including CRP, apha-2-macroglobulin and haptoglobin, in patients with MDD. Sluzewska et al. [21] reported increased serum CRP levels in association with other APP markers, including increased IL-6 and sIL-6R levels, and the alpha 1-acid glycoprotein microheterogeneity coefficient in patients with MDD compared with controls. Furthermore, the levels of transcortin (or corticosteroid-binding globulin), the primary glucocorticoid-binding protein and a negative control protein, were substantially lower in IMDD patients than in controls [39].

Substantial correlations were identified between changes in plasma haptoglobin levels and increased production of IL-6, as well as increases in the absolute counts of leukocytes, neutrophils, monocytes, and T cells that express T-cell activation markers, such as CD25+ and HLA-DR+ markers [40]. These results suggested that the modifications in APPs in MDD are the result of an increase in the production of proinflammatory cytokines, including IL-6 and IL-1β [27]. The mean polymorphonuclear elastase level was found to be significantly greater in patients with depression, especially in patients with MDD, than in healthy subjects [41].

Notably, the acute phase of mania and major depressive episodes (MDEs) in bipolar disorder are accompanied by an AP or inflammatory response and IRS activation [19, 42, 43]. This is due to the increased levels of cytokines that induce characteristic alterations in positive and negative APPs in the liver [44].

## Interactions among NIMETOX pathways in MDD

### Immune‒lipid interactions

Clinical trials that reduced serum cholesterol levels in patients with coronary heart disorder were counterbalanced by a substantial increase in suicide, as evidenced by early studies conducted in the 1990s [45–47]. In the 1990s, a few publications demonstrated that decreased serum cholesterol levels are associated with MDD and mania [48, 49]. Additionally, Morgan et al. [50] reported an inverse relationship between the severity of depression and the serum cholesterol concentration.

An initial study that demonstrated the involvement of immune‒metabolic interactions in MDD reported substantial correlations between decreased high-density lipoprotein cholesterol (HDL-C) and various indicators of immune activation [51]. The latter study was also the first to report an increase in atherogenicity in MDD patients using measurements of total cholesterol/HDL-C (Castelli risk index 1). Furthermore, IMDD is associated with a reduced degree of esterification of serum cholesterol, as determined by the formula (1—free cholesterol/total cholesterol) x 100 ^34^. This ratio reflects the activity of lecithin cholesterol acyl transferase (LCAT, EC 2.3.1.43), an enzyme bound to HDL-C particles [52, 53]. HDL-C transports excess free cholesterol as cholesteryl esters from peripheral tissues and macrophages to the liver through so-called reverse cholesterol transport (RCT) [53]. Reduced RCT activity may result in the accumulation of free cholesterol in the periphery, including in arterial walls and macrophages, which can contribute to atherosclerotic processes, inflammation and oxidative stress [53]. Additionally, HDL-C is a potent antioxidant that safeguards against the formation of reactive oxygen species (ROS) and prevents the oxidation of low-density lipoprotein (LDL), which is implicated in atherosclerosis [52, 53]. Consequently, the reduced activity of HDL-C and LCAT in MDD indicates decreased antioxidant potential and increased susceptibility to atherogenic processes, lipid peroxidation, IRS activation, and LDL oxidation.

These findings may elucidate the elevated autoimmune response in IMDD patients, in which phospholipids, including anticardiolipin, antiphosphatidylserine, and antipartial thromboplastin autoantibodies, are targeted [54, 55]. How such antibodies are generated in MDD became evident only two decades later, as will be discussed in one of the next sections. Cardiolipin, phosphatidylserine, and partial thromboplastin are phospholipids that are essential for cell membrane architecture and lipoprotein function. Cardiolipin is a phospholipid found in cell membranes, and phosphatidylserine safeguards neuronal cells in the central nervous system (CNS) and is crucial for neurocognition, whereas partial thromboplastin is involved in the intrinsic pathway of blood coagulation [54, 55]. Elevated concentrations of these autoantibodies are observed in autoimmune diseases and correlate with a heightened risk of subclinical atherosclerosis and subsequent cardiovascular events [56].

Interactions between lipids and the immune system in MDD are further corroborated by evidence indicating that MDD is associated with reduced levels of ω3 polyunsaturated fatty acids (PUFAs) and in phospholipid and cholesteryl fractions, as well as in the red blood cell (RBC) membrane of affected individuals [57–59]. Hibbeln [60] reported that the annual incidence of serious depression was significantly correlated with increased fish consumption (rich in ω3). Investigations have indicated that these ω3 modifications are induced by heightened oxidative stress, resulting in damage to the double bonds of the PUFAs, as indicated by a diminished oxidative potential index in MDD [57, 58].

Moreover, the reduction in ω3 PUFAs and the decreased ω3/ω6 ratio were significantly and inversely correlated with decreased levels of a negative acute-phase reactant, specifically zinc [58]. Zinc serves as a potent antioxidant and a cofactor for enzymes (elongase enzymes and desaturases) essential for the elongation and desaturation of PUFAs [58]. ω3 PUFAs play a key role in MDD because of their anti-inflammatory properties, which are mediated by a) diminished synthesis of proinflammatory cytokines, including TNF-α, IL-1β, and IL-6; b) enhanced generation of specialized proresolving mediators; and c) competition with arachidonic acid (AA) production, resulting in a reduction in arachidonic acid-related eicosanoid synthesis [61].

Furthermore, in the 1990s, the concentrations of the fat-soluble antioxidant vitamin E were markedly lower in individuals with IMDD than in controls, and the reduction in vitamin E levels was inversely correlated with IRS activation [62]. Vitamin E mitigates ROS levels and safeguards against lipid peroxidation and the oxidation of ω3 PUFAs [62]. This paper also reviews additional antioxidants diminished in MDD, including albumin, zinc, tryptophan, tyrosine, and glutathione. Evidence of heightened lipid peroxidation was identified in the early 2000s [63–65].

In summary, aberrant lipid metabolism in MDD is correlated with IRS activation, heightened oxidative stress, and pro-atherogenic mechanisms, contributing to the comorbidity between MDD and cardiovascular disorders [61].

### Immune-protein and amino acid metabolism interactions

The cytokine-induced acute phase response in the liver results in a disturbance of protein metabolism, prompting the liver to generate elevated levels of APPs that assist in combating infections and injuries, facilitating tissue repair, restoring homeostasis, and exhibiting anti-inflammatory properties (e.g., C-reactive protein, haptoglobin, and alpha-1-antitrypsin) as well as antioxidant functions (e.g., haptoglobin, ceruloplasmin, and alpha-1 acid glycoprotein). Consequently, numerous beneficial APPs facilitate immunoregulatory functions that aid in reestablishing homeostasis [44]. The expression of other proteins, specifically negative APPs such as albumin and transferrin, decreases during IRS activation when the liver reallocates resources to synthesize positive APPs [44]. Moreover, IRS activation augments the loss of albumin from the bloodstream and the rate of albumin catabolism.

Consequently, reductions in negative APPs, including albumin, may lead to decreased total serum protein in MDD patients, which is correlated with alterations in the principal electrophoretically separated protein fractions and is indicative of IRS activation [66]. In the same study, the alpha-1 and alpha-2 fractions significantly increased, indicating a transition from albumin synthesis to positive APPs associated with alpha-1 (alpha-1 antitrypsin, alpha-1 acid glycoprotein and alpha-2 fractions (haptoglobin, ceruloplasmin, and alpha-2 macroglobulin)).

During the 1990s, the dominant theory regarding MDD remained the monoaminergic theory, particularly the serotonin hypothesis of MDD. A critical biomarker of MDD reflecting serotonergic dysfunction is diminished serum levels of L-tryptophan, the precursor of serotonin [67]. Nonetheless, decreased L-tryptophan levels are correlated with indicators of impaired protein metabolism and IRS responses in MDD patients, including decreased serum zinc, transferrin, and high-density lipoprotein cholesterol (HDL-C) levels and elevated leukocyte counts and CD4 + /CD8 + T-cell ratios [68, 69]. These findings indicate that the reduced availability of L-tryptophan in severe IMDD is a secondary effect of a more generalized disorder in protein metabolism and the IRS response mediated by cytokines. Notably, interferons, as well as IL-2, IL-1, and IL-6, may induce indoleamine-2,3-dioxygenase (IDO), the enzyme responsible for the catabolism of tryptophan into tryptophan catabolites (TRYCATs) [18].

This route is crucial for the metabolic regulation of the IRS response and the reestablishment of homeostasis. Initially, it represents a significant immunosuppressive, anti-inflammatory, and antioxidant pathway [70]. Second, the heightened metabolism of L-tryptophan, resulting in decreased tryptophan levels, provides supplementary protective benefits through the inhibition of T-cell growth through the deprivation of immune cells. Third, numerous TRYCATs possess anti-inflammatory properties, for instance, through the activation of pro-apoptotic pathways [70]. A reduction in tryptophan in antigen-presenting cells (APCs) is correlated with heightened immunoregulatory activities, including the recruitment of regulatory T (Treg) cells and the cytokine IL-10, which serve an immunoregulatory function [71]. Consequently, IDO activation results in Treg activation and the suppression of effector cells, serving as a major mechanism in the inhibition of bacterial and viral infections [70]. Tryptophan deprivation results in diminished proteosynthesis and decreased activation of the rapamycin pathway, perhaps contributing to polyneuromyopathy [71]. This metabolic control of the IRS response safeguards against hyperinflammation and facilitates immunological tolerance [71].

### Immune-erythron-iron metabolism interactions and nutritional immunology

In addition to immune-lipid, immune-protein, and immune-protein interactions, MDD is characterized by alterations in immune-iron metabolism and immune-erythron (RBC functions) interactions; consequently, compared with normal controls, patients with IMDD are characterized by significantly lower hematocrit and hemoglobin levels and a lower number of red blood cells [5]. Additionally, those with IMDD have substantially lower serum iron and transferrin levels and higher ferritin and soluble transferrin receptor (sTfR) serum levels [19, 21]. Moreover, these aberrations were significantly associated with IRS activation, suggesting that the IRS response may be responsible for the development of these metabolic disorders in MDD [5].

Erythron changes are thought to result from anemia of chronic disease or anemia of inflammation, which is common in diseases with protracted IRS activation [5]. Proinflammatory cytokines and hepcidin induce the latter, which reduces the viability of red blood cells in the bloodstream and inhibits erythropoiesis [5]. IRS activation is known to induce functional iron deficiency, a condition in which iron is redistributed to macrophages [5]. IL-6 and IL-1β may stimulate hepcidin in the liver during the AP response, thereby promoting iron retention and blocking dietary iron import. Consequently, serum iron levels are reduced as a result of reduced iron assimilation and macrophage iron retention, whereas total body iron remains unaffected [72].

Trace elements, such as iron and zinc, are sequestered by the host organism as part of regulatory “nutritional immunity”, which is designed to reduce pathogenicity during infection by starving pathogens of trace minerals [73]. The quantity of iron and zinc required for the proliferation of invading gram-negative, gram-positive bacteria and viruses is thereby restricted by IRS processes. Furthermore, as discussed above, lower levels of zinc may have caused the lower ω3 PUFA levels observed in MDD [58].

Another host defense mechanism involves the elevation of ferritin, which limits the availability of iron [5, 74]. The primary iron reservoir in the body is transferrin, a negative acute phase protein, which facilitates the movement of iron throughout the circulation to diverse tissues [75]. The transferrin receptor facilitates iron uptake from transferrin into cells, but the soluble transferrin receptor is detectable in serum following its cleavage from the membrane-bound receptor [74]. Elevated serum soluble transferrin receptor levels indicate a reduction in body iron status, the concentration of membrane transferrin receptors, and IRS activation [74]. Thus, iron deficiency anemia resulting from IRS activation is largely indicated by increased soluble transferrin receptor levels in MDD patients.

### Immune–hypothalamic–pituitary–adrenal (HPA) axis interactions

The elevated activity of the HPA axis is a significant element of the homeostatic response, including during MDD. The primary function of the HPA axis is to release glucocorticoids, which regulate immune responses, basal metabolism, and salt and water balance, thereby mediating the effects of internal and external stressors. In the 1970s–1980s, MDD was associated with an increase in the activity of the HPA axis, as evidenced by increased basal cortisol levels, free urinary cortisol levels, increased ACTH and beta-endorphin levels, and the escape of these hormones from the suppressive effects of dexamethasone [13, 76]. Chronic hypercortisolemia may induce cognitive impairments and immunosuppression, in addition to activating the amygdala and causing neuronal damage in the hippocampus [77]. In MDD, a substantial inverse correlation was observed between immune function tests (LPA tests) and signs of increased HPA axis [14].

However, in the context of IRS responses, the activation of the HPA axis may occur as a result of proinflammatory cytokines, including IL-1β, IL-6, and TNF-α. The HPA axis is modulated by feed-forward mechanisms that involve peripheral inflammatory factors, which may stimulate the HPA axis to increase cortisol production, which in turn exerts anti-inflammatory effects, thereby maintaining homeostasis [78]. In severe IMDD, significant associations were observed between postdexamethasone cortisol values and increased production of IL-1β and IL-6 [17, 79]. Furthermore, substantial positive correlations were detected between serum IL-6 and sIL-2R levels and plasma cortisol levels in severe MDD patients [80]. These results suggested that the HPA axis may be stimulated by increased inflammatory signaling and T-cell activation in MDD, which could subsequently result in immunosuppressive effects, as detected by the LPA test [14].

However, the relationships between IRS activation and HPA axis function in severe MDD are even more intricate. Lowy et al. [81] reported that the administration of dexamethasone was capable of suppressing LPA test results in normal volunteers, whereas there was partial escape from dexamethasone suppression in MDD patients. Moreover, MDD patients exhibit dexamethasone nonsuppression in the production of IL-1β and sIL-2R, whereas in normal volunteers, dexamethasone administration substantially reduces the production rate [16]. This phenomenon can be explained by the fact that activated lymphocytes are less susceptible to glucocorticoid inhibition [82, 83]. Moreover, glucocorticoid-induced inhibition of monocyte-dependent proliferative responses may be prevented by IL-2 administration [84], and IL-1 may counteract the inhibitory effects of glucocorticoids on purified T cells [85]. Consequently, the more generalized glucocorticoid resistance in severe MDD is largely attributed to the increased IL-1β and IL-2 signaling in that condition [27]. It was later discovered that glucocorticoid receptor dysfunction is involved in MDD [86, 87]. Since IRS activation is accompanied by glucocorticoid resistance, decreased negative feedback on the axis, and increased cytokine stimulation of the axis, one may conclude that increased cortisol activity in severe MDD is at least partially secondary to IRS and T-cell activation [27].

### Etiologic factors in MDD: effects of acute and chronic psychological stressors

A notable characteristic of MDD is the array of etiological factors that contribute to its manifestation, as well as the various triggers associated with its onset [88]. These factors include psychological stressors, genetic predispositions, viral infections or reactivations, dietary influences (such as insufficient ω-3 polyunsaturated fatty acids and high-fat diets), and bacterial translocation [88]. Research in the 1990s initially concentrated on the impact of psychological stressors on the human immune system. Prior to this, chronic unpredictable mild stress in animal models, which simulates unforeseen negative life events in humans, has been shown to provoke peripheral immune response system activation, as previously reviewed [89].

Glaser et al. [90] reported that stress resulted in an increase in the accumulation of IL-2 in culture supernatant, which was accompanied by a downregulation of IL-2R expression and IL-2R messenger RNA levels. Dobbin et al. [91] demonstrated that examination stress significantly increased the production of IL-1β, whereas the production of IFN-γ and lymphocyte proliferation tests decreased [92, 93]. Maes et al. reported that stress stimulates the production of TNF-α, IL-1RA, IL-6, IFN-γ, and IL-10, as well as Th1 polarization, T-cell activation (CD2 + CD69+ and CD2 + HLA-DR + T cells), and B-cell proliferation (CD19 + ) [94].

Furthermore, psychological stress was associated with decreased albumin levels; elevated alpha-1, alpha-2, beta, and gamma protein electrophoresis fractions47; lower levels of CC16; and increased levels of sIL-6R and sgp130, suggesting a diminished anti-inflammatory capacity in the serum [46]. The severity of stress-induced anxiety, perceived tension, and distress was correlated with these alterations in PBMCs. Individuals with diminished serum ω3 PUFA levels or ω3/ω6 ratios have significantly heightened TNF-α and IFN-γ responses to psychological stressors compared with those with elevated serum ω3 PUFAs and ω3/ω6 ratios, respectively [95].

The administration of an acute stressor to patients with multiple sclerosis (MS) resulted in increases in the levels of IL-1β, TNF-α, and IFN-γ, whereas the level of IL-4 remained unaffected [96]. Crevicular IL-1β levels are markedly elevated in response to academic examination stress [97]. In a separate study, the production of IL-1β, IL-6, and IL-10 substantially increased in response to academic stress, whereas IFN-γ production decreased [98]. LPS-induced IL-6 production is further enhanced by stress [99]. Steptoe et al. [100] reported that serum IL-6, TNF-α, and sIL-1RA concentrations increased in correlation with systolic or diastolic blood pressure or pulse rate following stressor tasks [100].

A preliminary meta-analysis revealed that psychological stressors, such as speech stress, mental effort stress, mental arithmetic, and the Stroop test, increase the number of NK cells and cardiovascular metrics, including heart rate and systolic (SBP) and diastolic (DBP) blood pressure [101]. Another meta-analysis indicated that stress increased the counts of monocytes, neutrophils, and B cells, as well as IgG levels, while it decreased T-cell proliferation, IL-2R expression, and NK cell activity [102]. Chronic occupational stress in nurses was linked to immune dysfunction, as evidenced by immune activation (elevated counts of cells expressing IL-2R, particularly CD4 + CD25+ cells) and potentially immune suppression (a reduction in the percentage of natural killer cells). The elevation of activation markers, CD3 + CD16CD56+ cells, and serum neopterin was most significant in the high-stress/low-psychopathology group, whereas the reduction in CD8 + CD11b+ cells was most notable in the high-stress/high-psychopathology group [103].

### Clinical features or phenotypes of depression

During the 1990s, deep phenotyping was entirely unfamiliar and was further developed only three decades later. During the 1990s, case‒control studies were performed utilizing MDD based on the DSM or ICD criteria as the independent variable, with biomarkers serving as the dependent variables. Nevertheless, efforts have been made to associate the latter with clinical characteristics such as illness severity, melancholic features, suicidal tendencies, and distinct clusters of depressive symptoms. Indeed, as elaborated below, comprehensive clinical phenotyping is crucial for understanding and exploring the origins and pathophysiology of MDD. Nonetheless, the discoveries in the 1990s served as the foundation for the advancement of comprehensive clinical phenotyping.

The severity of illness, as assessed by the Hamilton Depression Rating Scale, was significantly inversely correlated with serum zinc levels; levels of transcortin [39]; serum tryptophan concentrations of [6, 27, 39]; leukocyte, neutrophil, and monocyte counts; haptoglobin levels; and serum concentrations of IL-6, sIL-1RA, and plasma neopterin [27, 104, 105].

The diminished concentrations of negative acute phase proteins, such as albumin and transferrin, exhibit excellent sensitivity (72%) and specificity (92%) for severe IMDD with melancholic features [36]. Furthermore, T-cell activation in patients with severe IMDD was notably heightened among inpatients with melancholia, and the numbers of pan-T, pan-B, T suppressor/cytotoxic, and CD21 + B cells significantly increased in patients with melancholia [24, 25]. Reduced levels of L-tryptophan are significantly observed in patients who exhibit melancholic features [27]. Furthermore, decreased saccharin preference (anhedonia), which is a key symptom of melancholia, was induced by the administration of LPS in a rodent model of depressive-like behavior [106]. These rodent behaviors are affected by antidepressant administration.

The first study on the correlation between immunological activity and suicidal behaviors was published in 1993 [107]. These authors reported that medication-free suicide attempters, primarily those with mood disorders, exhibited significantly elevated serum sIL-2R concentrations, indicating T-cell activation. Maes et al. [108] reported that the seasonal (including annual) variations in L-tryptophan, CD20 + B cells, and the CD4 + /CD8 + T-cell ratio serve as predictors of violent suicide [108]. Serum HDL-C levels are markedly lower in severely depressed men with a history of serious suicide attempts than in those without such behaviors [51]. T cells in suicidal depressive individuals exhibit predominantly Th1 characteristics, whereas T cells in nonsuicidal depressed patients display Th2 characteristics [109].

The initial papers that established the correlation between IRS activation and increased resistance to antidepressant treatment were published in the 1990s. For instance, treatment-resistant depression is defined by elevated serum levels of IL-6 and sIL-1RA [31] and decreased serum concentrations of zinc and L-tryptophan [6, 68].

### MDD and sickness behavior

Vegetative symptoms of melancholic depression, such as anorexia, weight loss, psychomotor retardation, sleep disorders, and anergy, are substantially and positively correlated with signs of the AP response, including increased serum haptoglobin levels and the alpha 2 globulin fraction [28, 110]. Anorexia, weight loss, reluctance to engage in social interactions, lethargy, inactivity, malaise, sleepiness and hyperalgesia are among the symptoms or sickness behaviors at the onset of febrile infectious disease [111, 112]. Nevertheless, other typical symptoms of melancholia, such as diurnal variation, early morning awaking, suicidal behaviors, and feelings of remorse, are not characteristics of sickness [112]. Additionally, the illness behavior complex is a well-organized, short-lived condition that is beneficial and lasts for approximately two weeks [112]. It allows inflammation-induced fever to contribute to the fight against bacterial and viral infections [111]. The sickness behavioral complex aids in the redirection of body resources to the high-energy demands of immune activation and fever in the fight against infections [111, 112]. In other words, the illness behavior complex enables the organism to undergo physiological and metabolic changes that improve its capacity to resist infection [112]. Unipolar MDD and bipolar major depressive episodes are, however, not short-term (2 weeks) conditions. In terms of sickness behavior, IRS activation has a beneficial function, whereas during MDE episodes, numerous NIMETOX pathways have a variety of detrimental effects, including anemia of chronic inflammation, increased oxidative stress with lipid damage, autoimmune responses to lipid fractions, disruptions of protein metabolism with reduced L-tryptophan availability to the brain, and disruptions of lipid metabolism with increased atherogenicity. As discussed below, the cumulative effects of the NIMETOX pathway lead to a reduction in neuroprotection and an increase in NIMETOX-associated neurotoxicity.

### Conclusions 1990–2000

**Fig. 1:**
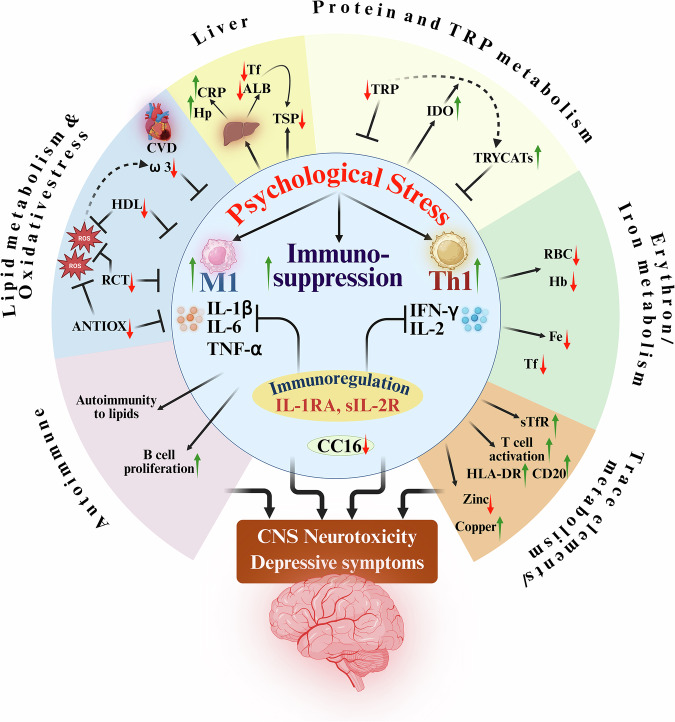
Knowledge regarding the interactions between neuroimmune, metabolic, and oxidative stress pathways increased between 1990 and 2000. Alb albumin, Antiox antioxidant, CC16 Clara cell protein, CNS central nervous system, CRP C-reactive protein, CVD cardiovascular disorder, Fe iron, Hb hemoglobin, Hp haptoglobin, IDO indoleamine-2,3-dioxygenase, IL-1R IL-1 receptor, IFN interferon, M1: M1 macrophage, RBC red blood cells, RCT reverse cholesterol transport, sTfR soluble transferrin receptor, Tf transferrin, Th1 T helper 1, TNF tumor necrosis factor, TRP tryptophan, TRYCATs tryptophan catabolites, TSP total serum albumin

The knowledge regarding the NIMETOX pathway interactions that accumulated between 1990 and 2000 is illustrated in Fig. 1. Maes [27] and Connor and Leonard [113] reported that MDD is associated with increased levels of proinflammatory M1 cytokines (including IL-1β, IL-6, and IFN-γ) and Th1-associated cytokines (including IL-2 and IFN-γ). Additionally, these cytokine aberrations in severe IMDD could elicit depressive symptoms; alter lipid, protein, and tryptophan metabolism; and induce nutritional immunity with lower levels of zinc and iron [27]. Notably, lower levels of these trace elements and L-tryptophan may contribute to depression symptoms in MDE patients.

Research conducted in the 1990s indicated that increased cytokine production plays a significant role in the mechanisms through which psychological stress can lead to MDD [113]. Nevertheless, only in the past twenty years have we gained a comprehensive understanding of the specific psychological stressors that are most influential in modulating the IRS/CIRS ratio in relation to MDD, namely, adverse childhood experiences (ACEs), as explained below.

It is crucial to emphasize that the majority of the findings were obtained in IMDD patients and patients with severe MDE and mania in BD and that the aberrations in these pathways were most pronounced in patients with melancholic depression. The clinical phenotype of the acute phase would prove to be of paramount importance in the assessment of the patient’s immune status only three decades later. Additionally, the acute phase of MDD is characterized by immunosuppression and activation of the IRS and CIRS. Three decades later, we demonstrated that imbalances in the IRS/CIRS are significant risk factors for various MDD phenotypes.

Licinio and Wong proposed that the activation of IRS represents a highly plausible mechanism to elucidate the fluctuating nature of depression, characterized by episodes of severity that may be succeeded by periods of partial or complete remission [114]. Merely two decades later, it became evident that the trajectory of illness serves as one of the most significant modifiers of the NIMETOX pathways, as well as the clinical phenomenology associated with MDD. In the 1990s, a significant proposition emerged regarding the role of cytokines within the CNS. These cytokines may modulate the biological underpinnings of depressive symptoms and regulate stress-responsive systems, as noted in the works of Kronfol and Remick [115] and Licinio and Wong [114]. Most importantly, these cytokines are involved in both neurotoxicity and neuroprotection [114]. Merely two decades later, it became evident that the equilibrium between neurotoxic and neuroprotective forces constitutes the most significant factor in the etiology of MDD, with numerous NIMETOX pathways playing a role in the heightened immune-linked neurotoxicity ratio and in lowered neuroprotection.

## Current knowledge of NIMETOX pathways based on findings between 2000 and 2026

### The era of systematic reviews and meta-analyses

Numerous meta-analyses have demonstrated that various components of the NIMETOX system exhibit aberrations in individuals diagnosed with MDD. Initially, a substantial body of reviews and overlapping meta-analyses indicated that MDD is associated with elevated levels of proinflammatory cytokines (e.g., IL-6, TNF-α, sIL-2R, and IL-1β) and APPs (e.g., CRP and haptoglobin), corroborating findings from the 1990s. Furthermore, these analyses revealed additional proinflammatory cytokines, specifically IL-12 and IL-18 [116–121], in MDD. These findings indicate an increase in the quantities of white blood cells, including neutrophils, granulocytes, monocytes, and natural killer cells, along with a heightened neutrophil/lymphocyte ratio and an increase in activated T cells [122, 123]. Furthermore, increases in the gene expression of proinflammatory cytokines and chemokines, such as CCL2, as well as immunoregulatory cytokines, including IL-10, have been observed in patients with MDD [124]. The significance of these findings for the immune-linked neurotoxicity/neuroprotective theory of MDD cannot be overlooked, as numerous proinflammatory cytokines—such as IL-1β, IL-6, IL-2, IL-8, IFN-γ, and IL-17—along with various chemokines, including CCL2, CCL5, and IP10, have been demonstrated to exhibit neurotoxic effects. These effects have the potential to damage neurons or disrupt their functional regulation [2].

Two meta-analyses indicated that MDD is characterized by antioxidant defenses alongside elevated biomarkers of oxidative stress [125, 126]. The findings corroborated that the levels of antioxidants, such as albumin, HDL-C, zinc, and total antioxidant capacity, were decreased, whereas serum peroxides and free radicals were elevated in individuals with MDD compared with those in the control group [126]. Moreover, the levels of vitamin C and paraoxonase 1 (PON1) activity were markedly lower in individuals with MDD than in control individuals [126]. PON1 is an antioxidant enzyme bound to HDL particles, and it plays a significant role in determining the antioxidant, anti-inflammatory, and anti-atherogenic properties of HDL-C [127].

Liu et al. [126] reported that the levels of oxidative damage products, such as malondialdehyde (MDA) (in both red blood cells and serum) and 8-F2-isoprostanes, were elevated in patients with MDD compared with control subjects. Furthermore, elevated concentrations of advanced oxidation protein products (AOPPs) were observed in patients with severe MDD, suggesting a detrimental effect on proteins attributable to chlorinative stress [128]. Other findings have indicated increased oxidative damage to DNA and a reduction in the levels of various antioxidants, including thiols (-SH), catalase, uric acid, glutathione, glutathione peroxidase, and ferric-inducing antioxidants [7, 125, 129, 130]. Notably, secondary impairment of mitochondrial functions, including the electron transport chain, may be evident in MDD [131].

Recent meta-analyses indicate heightened atherogenicity in MDD, characterized by an elevated Castelli risk index 1 (total cholesterol/HDL-C), an increased atherogenic index of plasma (triglycerides/HDL-C), a reduction in HDL-C levels, and a diminished RCT index [53, 132]. Another meta-analysis revealed that compared with healthy control subjects, individuals diagnosed with MDD exhibit diminished levels of ω3 PUFAs, specifically EPA and DHA [133].

A meta-analysis indicated that MDD is associated with diminished levels of L-tryptophan, which has a substantial effect size. However, the same study did not establish a correlation between MDD and the activation of IDO, and it was noted that kynurenine levels decreased [134]. The authors in question concluded that the reduction in tryptophan in MDD is not a result of IDO activation. Rather, it is likely attributable to decreased levels of albumin, which serves as the primary transporter of tryptophan in plasma. The subsequent depletion of L-tryptophan may shut off the TRYCAT pathway, thereby resulting in diminished TRYCAT values and concentrations, including those of kynurenine [134]. Conversely, the kynurenine/tryptophan ratio, which serves as an index of IDO activity, increased solely in patients with severe MDD, i.e., MDD with psychotic features [135]. Furthermore, consistent with the immune-linked neurotoxicity/neuroprotection theory, this meta-analysis revealed elevated levels of quinolinic acid, which is strongly neurotoxic, and decreased levels of kynurenic acid, which is neuroprotective, in MDD patients with melancholic and psychotic features [135].

## Novel key findings made between 2000 and 2026

### O&NS includes modified neoepitopes

Throughout this period, it became evident that ROS and RNS are instrumental in the initiation and progression of severe MDD. The principal outcomes associated with heightened production of nitric oxide (NO) include the formation of the neurotoxic substance peroxynitrite, as well as an increase in nitrosylation, which pertains to nitroso-binding to proteins [7]. The expression of the inducible nitric oxide synthase (iNOS) gene is significantly elevated in MDD patients [136]. Variations in the genes encoding iNOS and neuronal nitric oxide synthase (nNOS) have been associated with increased susceptibility to MDD [136].

Furthermore, secondary IgM-mediated autoimmune responses have been observed to target various nitroso-modified epitopes, including nitro-proteins, nitroso-tryptophan and nitroso-tyrosine [137, 138]. This research demonstrated that MDD is characterized by nitrosative aberrations, which include elevated synthesis of iNOS and nitric oxide (NO), increased nitroso binding to proteins or hypernitrosylation, and autoimmune reactions to the newly generated nitroso-adducts [7].

Similar secondary IgM-mediated autoimmune responses have been observed to target various oxidation-specific epitopes, such as MDA, azelaic acid, phosphatidylinositol, S-farnesyl-L-cysteine, and membrane anchorage molecules such as myristic and palmitic acid [138]. The responses in question may yield advantageous outcomes through the elimination of apoptotic cells, facilitated by the “eat me” signals that arise from elevated MDA expression on cellular membranes. Conversely, they may also produce adverse effects by disrupting the functionality of numerous proteins, including receptors that are tethered to membranes by the aforementioned anchorage molecules [138]. The development of these novel molecular entities and O&NS events may additionally contribute to atherosclerotic mechanisms, including IgG-mediated autoimmune responses targeting oxidized low-density lipoprotein [139]. The autoimmune responses in question are markedly elevated in patients with severe MDD and represent a principal element in the processes associated with atherosclerosis [139].

### IRS-CIRS measurements

The data mentioned in the previous sections indicate that immune parameters may have significant potential as diagnostic biomarkers for IMDD. Nonetheless, one must consider the various caveats that accompany their application as biomarkers or therapeutic targets in MDD. First, the immune biomarkers in question are influenced by various confounding factors, including age, sex, the menstrual cycle, body mass index (BMI), seasonal variations, and comorbid conditions such as metabolic syndrome (MetS) and (preclinical) atherosclerosis [140]. Second, in 2026, within the context of the machine learning era, biomarkers that are singular in nature no longer exist. Moreover, we are now able to employ multiplex methodologies that concurrently assess 48 or more than 100 cytokines, chemokines, and growth factors. Such data should be employed to calculate indices of CIRS alongside the diverse IRS profiles, which encompass M1 macrophage, alternative M2, Th1, Th2, Th17, and chemokine and growth factor profiles [2, 3]. In the Electronic Supplementary File (ESF), Table 1 presents the methodology for computing the IRS and CIRS profiles as z unit-based composite scores. IRS comprises immune activator molecules characterized by M1, Th1, and Th17 profiles, along with certain chemokines and growth factors associated with M1 or Th1. In contrast, the CIRS profiles include M2, Th2, and Treg profiles, as well as specific protective growth factors, as shown in the ESF (Table 1a, b).

IRS profiles are engaged to mitigate the threats posed by viral and bacterial infections. In contrast, the M2, Th2, and Treg profiles exhibit negative immunoregulatory and anti-inflammatory characteristics, which contribute to the maintenance of immune homeostasis and the prevention of an excessive inflammatory response [2, 112]. Consequently, this system facilitates the estimation of the M1/M2 ratio (which allows us to assess pro- versus anti-inflammatory forces), Th1/Th2 ratio (which allows us to assess Th1 versus Th2 polarization), and IRS/CIRS ratio, once more employing z unit composite scores of [2, 3]. An elevated IRS/CIRS ratio reflects the net impact of IRS activation alongside the disruption of immune tolerance, whereas a diminished IRS/CIRS ratio suggests that immunosuppressive CIRS effects may be more pronounced. Moreover, this approach enables the computation of immune-linked neurotoxicity profiles, as we gained more knowledge regarding the cytokines and chemokines that exert neurotoxic effects in experimental studies. ESF (Table 1a) presents an illustrative example of an immune-linked neurotoxicity profile.

Adult patients with very severe IMDD exhibit activation of both IRS and CIRS immune profiles, characterized by heightened TNF signaling and IL-6 trans-signaling [2, 3]. Adolescents diagnosed with MDD exhibit a comparable profile characterized by heightened proinflammatory TNF-α signaling and IL-6 trans-signaling, which correlates with an increase in IL-10- and IL-4-mediated anti-inflammatory activity [141].

It is imperative to emphasize that the emerging IRS-CIRS profile in severe MDD markedly diverges from the characterization established in the 1990s, as well as from the mainstream paradigm, which predominantly involves inflammation (increases in IL-6, TNF-α, and CRP levels) in “inflammatory depression”. In summary, our findings indicate that the immune profile associated with severe IMDD is characterized by markedly elevated levels of IRS and CIRS profiles, along with an increased Th1/Th2 ratio and an immune-linked neurotoxicity profile. Conversely, no significant differences were observed in the Th2, Th17, M1/M2, or IRS/CIRS profiles between IMDD patients and control subjects [3]. Consequently, the IMDD is distinguished by Th1 polarization and by a novel homeostatic setpoint between activated IRS and CIRS profiles, culminating in a significantly heightened immune-linked neurotoxicity profile.

IL-1β and IL-6 are not the most significantly elevated cytokines in the acute phase of severe IMDD; rather, it is IL-16, a Th1 cytokine, along with TNF-related apoptosis-inducing ligand (TRAIL) and TNF-β. [3] This observation suggests that TNF signaling (TRAIL, TNF-α, and TNF-β) is a major component of severe IMDD. Furthermore, Almulla et al. [3, 53] demonstrated that elevations in the expression of chemokines such as CCL27 and CCL5, along with colony-stimulating factors (CSFs) such as macrophage colony-stimulating factor (MCSF), and growth factors such as stem cell growth factor 1 (SCGF1 or Kit-ligand), platelet-derived growth factor (PDGF), and hepatocyte growth factor (HGF), are significantly greater than the increases observed in the expression of IL-6 or IL-1β.

The significance of these cytokines and growth factors lies in their role in promoting T-cell activation or immune-linked neurotoxicity or neuroprotective effects. SCGF1 is instrumental in regulating cell survival, proliferation, and the maintenance of stem cells, whereas PDGF and HGF are recognized for their neuroprotective and healing properties. For instance, platelet-derived growth factor (PDGF) facilitates neuronal growth and differentiation, orchestrates the interactions between neurons and glial cells, and has the potential to mitigate excitotoxicity [142]. HGF enhances the functionality of the blood–brain barrier, safeguards neurons against apoptosis, promotes neuronal viability, suppresses CNS autoimmunity, and stimulates the induction of CD25 + FOXP3+ Treg cells [143]. Nevertheless, it is important to note that both growth factors also promote immune activation [3, 144]. It is evident that the IRS response in the IMDD is marked by a concurrent increase in CIRS activity along with neuroprotective factors, indicating the presence of an evolutionary process to prevent hyperinflammation and initiate the healing process [112, 144].

However, as will be elaborated upon in the subsequent sections, the majority of patients with MDD do not exhibit a classical IRS profile, as evidenced by elevated scores in M1, Th1, and Th17 or IRS profiles, although most MDD patients present with immune-linked neurotoxicity profiles.

### IRS sensitization in MDD

It is crucial to emphasize that our discoveries regarding the elevated production of IL-1β, IL-6 and IFN-γ in IMDD were derived from studies using culture supernatant from in vitro-stimulated PBMCs (see “Increased production of Th and macrophage cytokines”). The measurements in question partially represent the in vivo immune-stimulated state and facilitate the assessment of cytokines that were more challenging to quantify in serum in the 1990s [33]. Nonetheless, these assays primarily assess the extent of sensitization of the immune system subsequent to immune injury, such as the administration of in vitro LPS + PHA [144]. Consequently, it is possible to assess the production of cytokines under both unstimulated and LPS + PHA-stimulated conditions. The residualized values obtained after adjusting for unstimulated production serve to indicate the extent of sensitization to immune injury. In this context, it has been observed that individuals experiencing very severe MDD exhibit sensitization in their M1, Th1, Th2, Th17, Treg, IRS, CIRS, and immune-linked neurotoxicity profiles [144].

### Activated T-cell versus Treg profiles

As discussed in “Introduction”, as early as the 1990s, IMDD was associated with T-cell activation, as evidenced by flow cytometry and the presence of T-cell activation markers (q.v. T- “cell activation”). In 1995, a novel population of T cells that function to inhibit the immune system, specifically Treg cells, was identified [145]. In 2003, Treg cells were characterized more precisely, revealing that FOXP3 serves as the intracellular transcription factor responsible for the differentiation of Treg cells [146]. These studies have demonstrated that mice deficient in FOXP3 exhibit markedly heightened activity of T effector cells, along with an increased inflammatory response and a propensity for autoimmunity. Treg cells exert their regulatory and anti-inflammatory effects through the production of immunoregulatory cytokines, including IL-10, transforming growth factor-beta (TGF-β), and IL-35 [147]. The cytokines in question exhibit CIRS activities and possess the potential to inhibit M1 and Th1 polarization. Furthermore, Treg cells engage with dendritic cells, consequently inducing IDO, which demonstrates immunosuppressive activity, induces cytolysis and disrupts the metabolism of T effector cells [147].

In individuals with drug-free MDD, the frequency of FOXP3 and the production of TGF-β are notably lower than those in healthy volunteers [148]. Rachayon et al. reported imbalances between activated T effector cells, characterized by the presence of CD69 + , CD71 + , CD40L + , and HLADR+ on CD3 + , CD4 + , or CD8+ cells, and a comparatively reduced population of Treg cells, particularly those expressing CD152 or GARP (glycoprotein A repetitions predominant) alongside CD25 + FOXP3 markers. GARP serves as a surface molecule on Treg cells and is known to release TGF-^β4^.

Notably, the remission phase of MDE in bipolar disorder patients is characterized by a heightened prevalence of CD4 + CD25 + FOXP3 + CD152+ cells and a reduction in effector T cells [149]. Consequently, the acute phase of MDEs may be characterized by a heightened T effector/Treg cell ratio, whereas the remission phase is characterized by an increase in the number of Treg cells that produce TGF-β [149]. This finding suggests that the acute phase of illness is associated with heightened neurotoxicity resulting from elevated T effector activity and diminished Treg cell function. Conversely, the remission phase may be marked by enhanced healing processes facilitated by increased Treg activity [149, 150]. Nonetheless, further research is needed to investigate both the acute and remission phases of MDE.

### MetS and NIMETOX pathways

During the 2010s, various reports indicated a greater incidence of MetS among patients diagnosed with MDD, suggesting that the latter may increase the risk of developing MetS [151]. Consequently, MetS was identified in 27.9% of patients diagnosed with MDD. Notably, compared with individuals who presented with their first episode, individuals who experienced recurrent episodes had a higher prevalence of MetS (45.2%) [151]. The common pathways observed in mood disorders and MetS are characterized by low-grade inflammation, including the presence of elevated APPs and certain proinflammatory cytokines, an increased atherogenic index of plasma, and Castelli risk indices [152]. Additionally, there are decreased levels of HDL-C, PON1, LCAT and RCT and heightened lipid peroxidation and the formation of aldehydes such as MDA and oxidized LDL cholesterol under both conditions [152].

Furthermore, the increased atherogenicity observed in MDD and comorbid MetS is further exacerbated by reduced levels of apolipoprotein A1 (ApoA1), which serves as an additional antioxidant that safeguards HDL-C together with LCAT and PON1 from oxidative damage [53, 140]. These findings suggest that the HDL-C-PON1-LCAT-ApoA complex is a significant factor in MDD and its comorbidity with MetS [140]. Moreover, elevated levels of ApoB and ApoE play a significant role in fostering an atherogenic state associated with this condition [140]. A recent study demonstrated that MDD is characterized by elevated serum levels of oxidized HDL-C (oxHDL) and a significantly reduced HDL-C/oxHDL ratio [153]. These findings indicate increased susceptibility of HDL particles to oxidative modification, which compromises their functional properties and serum concentrations. Consequently, impaired HDL-C function leads to reduced RCT, promoting the accumulation of oxidized lipids and thereby amplifying oxidative stress and immune activation in MDD [153].

A study that simultaneously controlled for MDD, tobacco use disorder, MetS, and generalized anxiety disorder revealed that partially shared ROS/RNS, along with O&NS pathways, underlie the comorbidity of mood disorders, MetS, and generalized anxiety disorder [154]. Furthermore, MetS was independently associated with increased levels of AOPP and MDA, whereas atherogenicity and insulin resistance were significantly correlated with O&NS toxicity.

Notably, adiponectin, a protein hormone secreted by adipose tissue, serves as a significant antioxidant, the levels of which are diminished in MDD [155]. Numerous studies have indicated that leptin, a crucial hormone involved in the regulation of energy balance and BMI, may be involved in MDD. While an initial investigation into serum leptin yielded negative findings [156], subsequent meta-analytical research indicated that diminished levels of serum adiponectin and leptin (in males) are associated with an elevated risk of developing MDD [157].

### Toll-like receptor 4 (TLR4) and nuclear factor (NF)-κB

MDD patients exhibit elevated levels of NF-κB RNA and TLR-4 RNA and protein among their PBMCs [124, 158, 159]. Furthermore, the results of the aforementioned study revealed increases in the expression of TIR domain-containing adaptor molecule 1 (TRIF1), myeloid differentiation primary response gene 88 (MYD88), and TLR-3/4 genes in patients with MDD [124]. The baseline transcription levels of NF-κB factors serve as predictors for the onset of affective symptoms subsequent to the administration of LPS [160]. Pathogen-associated molecular patterns (PAMPs), including LPS, can activate TLRs, particularly TLR4. This activation subsequently initiates the NF-κB signaling pathway, which can result in the enhanced synthesis of type 1 interferon, proinflammatory cytokines, and ROS/RNS [161]. Notably, newly formed oxidation-specific epitopes have the potential to function as DAMPs, thereby activating the TLR4 complex. This activation can initiate an autoamplifying feedback loop, referred to as the TLR-Radical Cycle Pathway [161]. MYD88 and TRIF1, whose transcription is elevated in MDD, are integral to the signal transduction pathways associated with TLR4. Consequently, MYD88 functions as an adapter protein for TLRs and, through its interaction with the intracellular domain of TLRs, may facilitate the recruitment of additional signaling molecules, ultimately resulting in the activation of the NF-κB signaling pathway. TRIF serves as an adapter protein that plays a crucial role in the signaling pathways of TLR4, which is responsible for recognizing LPS, and TLR3, which detects double-stranded viral RNA. This interaction subsequently initiates the production of type I interferons, specifically IFN-α and IFN-β. Notably, the gene network analysis conducted on the cytokine, chemokine, and growth factor network associated with severe depression [144] revealed enrichment of hyperresponsive TLR4, NF-κB, and JAK/STAT pathways. These pathways are, in fact, significant contributors to peripheral IRS and O&NS responses [144].

It is important to recognize that both TLR4 and NF-κB are integral to the pathophysiology of MetS and atherosclerosis, with the latter potentially enhancing TLR signaling pathways. For instance, free fatty acids, such as palmitic acid, along with oxidation-specific epitopes, have the potential to activate TLR4 signaling pathways in PBMCs and adipocytes [161, 162]. TLR4 serves as a molecular connection between lipids and IRS, as well as O&NS responses, through which nutrition may influence the innate immune system’s responses that govern insulin resistance and energy balance [162]. Furthermore, TLR4 signaling plays a significant role in modulating adipocyte fat storage and disrupting adipogenesis and energy production, and it may exacerbate complications associated with obesity [163]. The signaling pathways associated with TLR4 significantly influence endothelial functions by enhancing vascular permeability and the expression of adhesion molecules. Furthermore, the activation of TLR4 in macrophages may play a role in the instability and rupture of plaques [164, 165].

The activation of NF-κB interferes with insulin signaling, which subsequently disrupts glucose and lipid metabolism. This interference enhances insulin resistance and exacerbates MetS, as noted by Baker et al. [166]. A portion of these mechanisms operates through the activation of IRS and O&NS processes. The activation of NF-κB is integral to the processes associated with atherosclerosis, as it influences endothelial dysfunction and facilitates the production of adhesion molecules [167, 168]. These processes contribute to the proliferation of plaques and their subsequent destabilization.

Increased free cholesterol [167] and hyperlipidemic dietary circumstances activate the NF-κB pathway in contrast to a normal diet [169], whereas high-fat diets may elicit depressive-like behaviors in animal models [170]. Importantly, the first episode of mild depression is accompanied by heightened indicators of preatherogenic processes, even in the absence of MetS [171]. Consequently, in patients experiencing their first episode, LCAT activity and RCT were diminished, while free cholesterol levels were elevated. Furthermore, those patients exhibited heightened indicators of O&NS, including elevated lipid peroxidation and aldehyde production [172].

Free cholesterol promotes atherosclerosis by diffusing into the arterial wall, accumulating and crystallizing within cells and macrophages, increasing cholesterol oxidation, inducing cytotoxicity, inhibiting membrane domain formation, and activating apoptotic pathways [173]. Consequently, it was posited that diminished RCT and the resulting lipid peroxidation constitute the principal pathways impacted during the acute phase of severe and mild MDD [171].

### NLRP3

The nucleotide-binding domain, leucine-rich repeat, and pyrin domain-containing protein 3 (NLRP3) inflammasome is a crucial complex in the innate immune system and endothelial cells, as well as in the CNS, including microglia [174]. Activation of the NF-κB pathway enhances the transcription of pro-IL-1β, pro-IL-18, and NLRP3, resulting in a primed inflammasome. MDD is associated with heightened NLRP3 inflammasome activity in PBMCs [175, 176]. In MDD, this process is characterized by mitochondrial malfunction, heightened oxidative stress, diminished antioxidant defenses, and elevated endoplasmic reticulum stress [175, 176]. Thus, the activation of the NLRP3 inflammasome plays a role in the activation of IRS and O&NS, potentially influencing the pathophysiology of MDD.

NLRP3 mRNA levels, together with IL-1β and IL-18 levels, are correlated with an increased risk of atherosclerotic disease [177]. Cholesterol crystals, oxidized phospholipids, intracellular stress signals, and NF-κB signaling contribute to the activation of the NLRP3 inflammasome [177]. Activation of NLRP3 subsequently stimulates M1 activation and exacerbates inflammation in adipose tissue and metabolic diseases [178].

### Bacterial translocation, gut dysbiosis and the gut–immune–brain axis

In 2008, it was demonstrated that MDD is associated with increased bacterial translocation of gram-negative bacteria, including *Pseudomonas putida, Pseudomonas aeruginosa, Citrobacter koseri, Klebsiella pneumoniae, Morganella morganii*, and *Hafnia alvei* [179]. A composite score based on IgM/IgA responses to gram-negative bacteria demonstrated significant diagnostic efficacy for MDD, with an area under the curve of 90.1%. This score was significantly correlated with gastrointestinal symptoms, fatigue, autonomic symptoms, and a subjective perception of infection [179]. These findings indicate that the increased translocation of LPS from gut-commensal gram-negative bacteria is due to increased intestinal permeability (or a leaky gut). Additional biomarkers indicative of a leaky gut are commonly noted in MDD, including alterations in zonulin, LPS-binding protein (LBP), and sCD14 [180, 181]. Leaky gut and metabolic endotoxemia may result from suboptimal diets high in lipids and sugars, chronic stress, IRS activation, elevated O&NS and gut dysbiosis [179].

The heightened LPS translocation identified in MDD is significantly correlated with IgG responses to oxidized LDL, IgM responses to various oxidation-specific epitopes (including MDA and azelaic acid), nitro adducts, and serum lysozyme [182]. Autoimmune reactions to oxidized LDL are critical factors contributing to atherosclerosis, and autoimmune responses to oxidation-specific epitopes may exacerbate atherosclerotic processes [182]. Lysozyme is a bacteriolytic enzyme that hydrolyzes bacterial polysaccharides and may diminish the endotoxic activity of LPS [183].

As discussed in the preceding section, LPS may stimulate the TLR4-NF-κB pathway, resulting in heightened O&NS and IRS activation. LPS increases the risk of atherosclerosis through various mechanisms [184]. Furthermore, LPS may induce vascular inflammation, increase the expression of adhesion molecules, and facilitate the migration of inflammatory cells to the site [184]. The primary receptors on inflammatory cells that mediate responsiveness to LPS and are linked to atherosclerosis are CD14, myeloid differentiation protein 2 (MD-2), and LBP [184]. Metabolic endotoxemia, characterized by elevated levels of LPS in the bloodstream, is correlated with insulin resistance, increased adiposity, and a heightened risk of MetS and obesity [185, 186].

Numerous studies have indicated that MDD is associated with gut dysbiosis, characterized by an increased prevalence of proinflammatory pathobionts, such as *Enterobacteriaceae* and *Eggerthella*, which may compromise gut wall integrity, as well as a diminished presence of anti-inflammatory probiotics, including *Ruminococcus* and *Faecalibacterium*, that enhance gut wall integrity [187–190]. Recently, a specific gut dysbiosis enterotype was shown to be associated with decreased HDL-C levels and increased atherogenicity in patients with MDD, namely, the protective effects of *Bifidobacterium, P. merdae* and *Romboutsia* and increased risk via *Proteobacteria* and *Clostridium sensu stricto* [187]. Furthermore, gut dysbiosis may be accompanied by decreased production of short-chain fatty acids, such as butyrate, which attenuate inflammation, improve gut epithelium integrity, and lower vitamin production by the microbiota [191].

Furthermore, diminished RCT, PON1, HDL-C, and ω3 PUFAs may eliminate or neutralize the LPS burden or signaling. The macrophage ABCA1 transporter, which modulates lipid transport, also participates in the elimination of LPS [192]. ω3 PUFAs may increase *Bifidobacteria* levels and reduce *Enterobacteria levels*, thereby mitigating metabolic endotoxemia [193, 194].

Most LPS is linked to HDL-C, which may therefore neutralize LPS, thereby inhibiting TLR4 signaling [195]. Furthermore, HDL-C promotes the release of LPS bound to macrophages, hence decreasing macrophage activation [195]. Moreover, ApoA1 independently neutralizes the toxicity of LPS by binding to the lipid A component of LPS. PON1 hydrolyzes N-acyl homoserine lactones that facilitate quorum sensing (cell-to-cell communication) in gram-negative bacteria, including *P. aeruginosa*. Consequently, decreased PON1 activity may be associated with heightened quorum sensing by *P. aeruginosa* [196]. Apart from bacterial translocation due to a leaky gut, new research has shown that microbiota in the dental-brain axis, uterus-brain axis, and skin-brain axis may be involved in MDD [128, 197, 198].

**Fig. 2:**
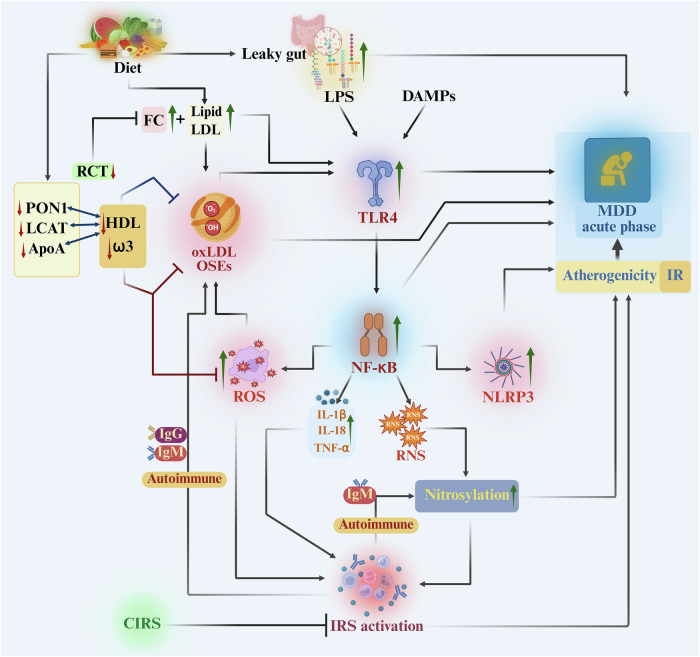
Central role of Toll-like receptors (TLRs) and nuclear factor (NF)-κB in the pathophysiology of major depressive disorder (MDD). ω3 Omega-3 PUFAs, ApoA apolipoprotein A, CIRS compensatory immunoregulatory system, DAMPs damage-associated molecular patterns, FC free cholesterol, HDL high-density lipoprotein, IL interleukin, IRS immune-inflammatory response, LCAT Lecithin–cholesterol acyltransferase, LDL low-density lipoprotein cholesterol, LPS lipopolysaccharides, NLRP3 NOD-like receptor protein 3 inflammasome, OSEs oxidative specific epitopes, oxLDL oxidized LDL, PON paraoxonase, RCT reverse cholesterol transport, RNS reactive nitrogen species, ROS reactive oxygen species, TNF tumor necrosis factor

The central role played by TLR4 and NF-κB in the pathophysiology of MDD is shown in Fig. 2. Activation of the TLR4 complex by DAMPs and LPS, resulting from increased intestinal permeability, initiates a cascade of processes that activate NF-κB, ROS/RNS, nitrosylation, IRS, NLRP3, and the creation of oxidation-specific epitopes, culminating in autoimmune responses to oxidation-specific epitopes and nitroso-adducts. Reduced activity of RCT, due to diminished levels of PON1, LCAT, and ApoA1, may lead to excessive accumulation of free cholesterol, resulting in the generation of oxysterols that could subsequently activate IRS and induce O&NS. Dietary patterns high in fats and low in ω3 PUFAs and diets that may compromise PON1 activity, gut wall integrity and gut microbiome homeostasis may exacerbate this sequence of events.

### Multicausal etiology

There is now substantial evidence that the heightened incidence of MDD in many medical diseases and conditions can be attributed to activated IRS and O&NS pathways [1]. Neurological comorbid illnesses such as MS, stroke, Alzheimer’s disease, Parkinson’s disease, and Huntington’s disease share common pathways involving CNS neuroinflammation and neurodegenerative processes. Systemic comorbid medical conditions share pathways involving peripheral IRS and O&NS pathways, e.g., chronic obstructive pulmonary disease, inflammatory bowel disease, rheumatoid arthritis, systemic lupus erythematosus, irritable bowel syndrome, psoriasis, type 1 and type 2 diabetes, HIV infection, obesity, and chronic fatigue syndrome [1]. Notably, medical conditions, including interferon-α-based immunotherapy, hemodialysis, and the postnatal period, are associated with a heightened risk of MDD, which is attributable to activated IRS and O&NS pathways [88].

Table 2 presents a compilation of the pathways described above, demonstrating that NIMETOX pathways are fundamental to the emergence of depressive symptoms in patients with concurrent medical conditions, such as MS and relapsing-remitting MS, transfusion-dependent thalassemia, atherosclerosis with unstable angina, type 2 diabetes mellitus, long COVID-19, preeclampsia, and end-stage renal disease.

**Table 2:** Associations between the neuroimmune, metabolic, and oxidative stress (NIMETOX) pathways and depressive symptoms due to comorbid disorders

	Disease	Biomarker	References
1	Long COVID	PGE2, CRP, Galanin/GALR1, IR/PAI1, hypoxia	[244]
2	Long COVID	IgM-HHV-6-dUTPase, IgG-HHV-6, IgA-activin-A, CRP	[245]
3	Long COVID	CRP, KYN/TRY (increased KYN and reduced TRY), increased IDO, insulin resistance, reduced SpO_2_ (hypoxia)	[246]
4	Long COVID	Neurotoxicity index (IL-1β, IL-18, CRP, MPO, AOPP), Lowered Ca, Lowered SpO_2_	[247]
5	Long COVID	Increased neuro-oxidative stress toxicity (increased MPO, NOx, zinc, MDA, and decreased Gpx), reduced SpO_2_ and increased PBT	[248]
6	COVID-19 infection	IRS + lung lesions, Lowered SpO_2_	[249]
7	Rheumatoid arthritis (RA)	DAS 28-CRP, Decreased BDNF	[250]
8	RA	*CXCL10, MARCO, PLA2G7, ERAP2, BTN3A2, AURKA*	[251]
9	RA	No causal relationship between depression and RA	[252]
10	RA	Immune-inflammatory pathways, Rheumatoid factor, Anti-citrullinated protein antibodies, CD17, Mu-opioid receptor	[237]
11	End-stage renal disease	Inflammation, copper, NFL, Nestin, S100B, MBP	[253]
12	Type 2 diabetes mellitus	Insulin resistance coupled with atherogenicity, lowered calcium. Increased β-arrestin-1, increased copper and increased LacCer	[254]
13	Atherosclerosis	IL-6, IL-10, Copper, Zinc, Mu-opioid receptor, calcium, unstable angina, Atherogenicity + insulin resistance	[255]
14	Transfusion-Dependent Thalassemia	Iron overload, increased IL-1β, and increased TNF-α	[256]
15	Multiple sclerosis (MS)	IL-10 (negatively associated)	[257]
16	MS	CD4^+^CCR7^low^T_CM_ cell frequencies, Th1-like phenotype	[258]
17	MS	Neuroinflammation, Peripheral inflammation, Gut dysbiosis, Oxidative and nitrosative stress, Mitochondrial dysfunction	[259]
18	MS	TNF-α, IFN-γ, IL-6, IL-10	[260]
19	MS	*TNFB1/B2* genotype, increased IL-6, lower IL-4, lower albumin	[261]
20	Relapsing-remitting MS	Activation of IRS, M1 macrophage, Th1, Th17, growth factor, and CIRS, Aberrations in the erythron	[262]
21	Inflammatory bowel disease (IBD)	Inflammation and immune regulation, *HGF, SPARC, ADAM12, MMP8*	[263]
22	IBD	Calprotectin	[264]
23	IBD	Inflammation, CRP/albumin ratio, albumin	[265]
24	Preeclampsia	Checkpoint molecules: sCD80/sCTLA-4, Vitamin D, Calcium, copper	[266]
25	Psoriasis	TNF-α increase, IL-17A increase, and IL-23 increase.	[267]
26	Alzheimer’s disease	Higher tau, Lower Aβ_42_	[268]
27	Parkinson’s disease	Decreased serum BDNF	[269]
28	Parkinson’s disease	Increased serum S100β	[270]

PGE2 prostaglandin E2, CRP C-reactive protein, GALR1 Galanine receptor 1, IR insulin resistance, PAI1 Plasminogen activator inhibitor-1, HHV human herpesvirus type 6, dUTPase deoxyuridine 5’-triphosphate nucleotidohydrolase, KYN kynurenine, TRY tryptophan, IDO indoleamine-2,3-dioxygenase, IL interleukin, MPO myeloperoxidase, AOPP advanced oxidation protein products, NOx nitric oxide metabolites, MDA malondialdehyde, Gpx glutathione peroxidase, PBT peak body temperature, DAS 28-CRP disease activity score 28-joint count C-reactive protein, BDNF brain-derived neurotrophic factor, RA rheumatoid arthritis, NF neurofilament light chain, S100B S100 calcium-binding protein B, MBP myelin basic protein, T2DM Type 2 diabetes mellitus, LacCer Lactosylceramides, TNF-α tumor necrosis factor-α, MS Multiple sclerosis, Th1 T helper-1, IFN-γ interferon-γ, RRMS relapsing-remitting multiple sclerosis, IBD inflammatory bowel disease, IRS immune-inflammatory response, *CIRS* compensatory immunoregulatory system

In 1998, it was established that ACEs markedly increase the likelihood of negative health outcomes in adulthood, such as MDD, BD, and cardiovascular disease [199]. Table 3 summarizes the effects of ACEs on NIMETOX pathways.

**Table 3:** Effects of adverse childhood experiences on neuroimmune, metabolic, and oxidative stress (NIMETOX) pathways in the general population (GEN) and major depression (MDD) patients

Sample	Biomarkers	Authors	PubMed ID
GEN	Von Willebrand Factor, C-reactive protein (CRP), fibrinogen	Lacey et al. [271]	32201253
MDD	Activation of the immune-inflammatory response system (IRS) pathways, lower compensatory immunoregulatory system (CIRS) activity	Maes et al. [272]	35563878
GEN	High interleukin (IL)-6, tumor necrosis factor (TNF)-α, and CRP	Wong et al. [273]	36177305
MDD	High activated T cell, low T regulatory cells	Maes et al. [150]	38945402
GEN	High atherogenicity indices	O’Leary et al. [274]	37090689
MDD	High atherogenicity, low reverse cholesterol transport	Maes et al. [275]	38246281
MDD	Higher IRS, M1 macrophage, T helper (Th)1, Th1 polarization, Th17, immune-linked neurotoxicity.	Almulla et al. [276]	38442479
MDD	Increased oxidative and nitrosative stress (O&NS) with increased lipid peroxidation and protein oxidation	Moraes et al. [277]	29542039
MDD	Lower Antioxidant levels (zinc, albumin, and-SH groups)	Moraes et al. [277]	29542039
GEN	High lipid peroxidation (isoprostane levels)	Horn et al. [278]	31026258
GEN	Hypothesis: Iron deficiency, IL-6 hepcidin-axis	Reid et al. [200]	39161875
GEN	High *Prevotella*	Hantsoo et al. [279]	30399404
MDD	Association with a specific gut dysbiosis enterotype highly specific for MDD	Maes et al. [187]	37052305
GEN	Impact gut microbiome, indicating possible gut integrity breakdown	Kazemian et al. [280]	38248533
GEN	Review: Amygdala responsivity, reduced gray matter volume	Hakamata et al. [201]	35331780

In the general population, ACEs may cause increased IRS activation, including increased APPs and cytokine levels (IL-6 and TNFα), proatherogenic profiles, oxidative stress, and gut dysbiosis in adulthood. A compelling theory posits that ACEs may influence iron metabolism, potentially resulting in iron shortage and IL-6 signaling, which are linked to neurodevelopmental consequences [200], and an amplified amygdalar response to emotionally unfavorable stimuli and a reduced amount of hippocampal gray matter [201]. In MDD, retrospectively evaluated ACEs are strongly correlated with heightened IRS activity, T-cell activation, Th1 and M1 polarization, immune-linked neurotoxicity, decreased CIRS activation and Teg cells, IRS sensitization, lipid peroxidation, protein oxidation, diminished antioxidant defenses, and gut dysbiosis.

Importantly, the apparent discrepancy between transient stress responses (described in “Etiologic factors in MDD: Effects of acute and chronic psychological stressors”) and the more persistent immune activation in MDD and immune sensitization due to ACEs is addressed within the NIMETOX framework by distinguishing different key processes. First, ACEs may drive long-standing sensitization of immune pathways, leading to a primed state [1]. Second, subsequent triggering factors, such as negative life events or ongoing stressors (viral, bacterial, metabolic, psychosocial), might reactivate these sensitized pathways, resulting in sustained or recurrent activation. Thus, the immune profile observed in MDD can be understood as the result of repeated reactivation of sensitized NIMETOX pathways by multiple hits rather than a simple extension of an acute stress response. This conceptualization explains both the similarities between stress-induced and MDD-related immune changes and their persistence over time.

### Peripheral NIMETOX pathways and neuroinflammation

**Fig. 3:**
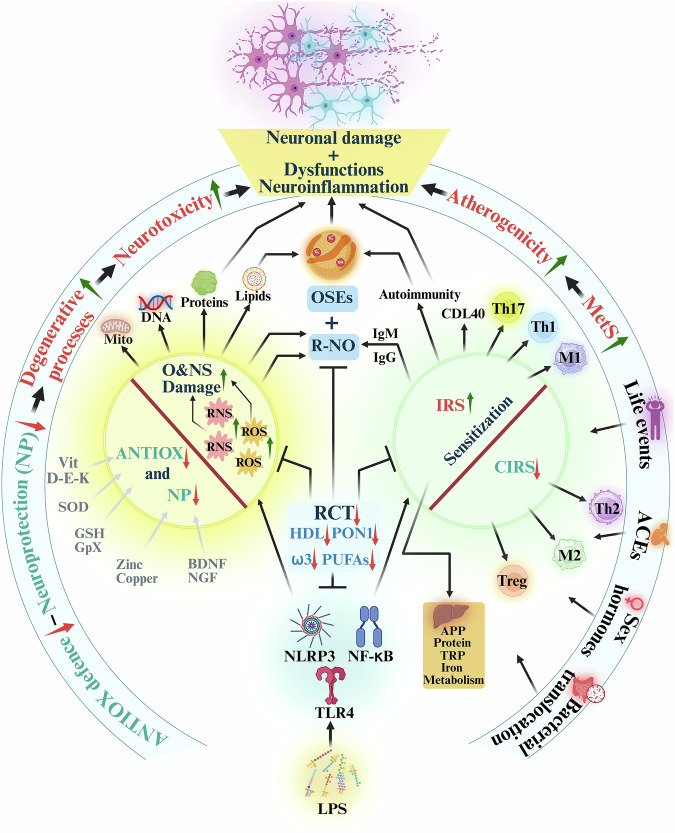
Various neuroimmune metabolic oxidative stress (NIMETOX) pathways are implicated in the acute phase of severe major depression. ω3 PUFAs polyunsaturated fatty acids, ACEs adverse childhood experiences, Antiox antioxidant, APP acute phase response, BDNF brain-derived neurotrophic factor, CIRS compensatory immunoregulatory system, GSH glutathione, Gpx glutathione peroxidase, HDL high-density lipoprotein, LPS lipopolysaccharides, M1/2 M1 (classical)/2 (alternative) macrophage, MetS metabolic syndrome, NGF nerve growth factor, NF-κB nuclear factor (NF)-κB, NLRP3 NOD-like receptor protein 3 inflammasome, O&NS oxidative and nitrosative stress. OSE oxidative-specific epitopes, PON paraoxonase, RCT reverse cholesterol transport, R-NO nitroso adducts, RNS reactive nitrogen species, ROS reactive oxygen species, SOD superoxide dismutase, Th T helper, TLR Toll Like Receptors, Treg T regulatory, TRP tryptophan

The various pathways implicated in the acute phase of severe MDD, including the LPS-induced expression of NF-κB and TLR4, as well as imbalances in IRS versus CIRS, O&NS versus antioxidant defenses and atherogenic versus anti-atherogenic factors, are shown in Fig. 3. Decreased LCAT, PON1 and ApoA1 defenses may lead to impaired RCT, which, together with decreased ω3 PUFAs, are associated with decreased antioxidant, anti-inflammatory and anti-atherogenic properties. The culminating effects of these pathways may ultimately cause neurotoxicity and atherogenic effects, leading to depressive symptoms. In this section, we review the evidence that these peripheral NIMETOX pathways may lead to neuroinflammation, microglial M1 activation, neuronal damage and functional deficits and that increased lipid load may impact these pathways.

Recent data indicate the presence of neuroinflammation and neurotoxic consequences in the CNS of patients with MDD [1]. This evidence emerged from studies utilizing positron emission tomography (PET) to evaluate the levels of the 18-kDa translocator protein (TSPO) expressed by activated microglia [202]. Postmortem analyses revealed neuroinflammation and M1 microglial activation, as evidenced by elevated proinflammatory cytokine, TLR3 and TLR4 mRNA expression and heightened oxidative stress [1]. Postmortem investigations of neuronal and glial cells in MDD revealed neuronal atrophy, neuronal shrinkage, an increased density of microglia, a reduction in astroglial cells, impaired neurogenesis, a decrease in hippocampal volume, a decreased density of total glia and oligodendrocytes in the amygdala, and a depletion of oligodendrocytes [1].

Peripheral levels of neuronal injury biomarkers reveal elevated serum levels of glial fibrillary acidic protein, neurofilament light chain, P-tau, and other anomalies that are indicative of myelination problems, astroglial damage, neuronal impairment, and synaptic dysfunctions [203]. Moreover, evidence of blood‒brain barrier disruption in MDD is shown by dynamic contrast-enhanced MRI [204]. This results from the impairment of blood–brain barrier tight junction expression, endothelial cells, and alterations to transport pathways caused by proinflammatory cytokines, LPS, toxic activated T cells (e.g., CD40L + ), ROS, O&NS, neurovascular dysfunctions, and increased free cholesterol levels [1, 205–208]. Consequently, neurotoxic cytokines, chemokines, colony-stimulating factors (e.g., MCSF), activated T cells (CD40L + ), LPS, TRYCATs, M1 macrophages, and free cholesterol may readily translocate from peripheral blood into the brain, resulting in neuroinflammation and damage to neuronal and glial cells [1].

It is crucial to recognize the substantial correlations in MDD between peripheral NIMETOX pathways and cerebral lesions and dysfunctions associated with MDD. Peripheral IRS activation, evidenced by increased plasma levels of TNF-α receptor subtype 1, IL-6, and IL-8, is significantly correlated with atypical brain metabolism, cortical atrophy, diminished cortical gray matter, compromised integrity of white matter tracts in mood-related neural networks, and altered functional connectivity and activation patterns of cerebral circuits [1, 209]. In MDD, elevated blood CRP levels and insulin resistance are significantly correlated with the levels of neuronal damage indicators, such as glial fibrillary acidic protein, neurofilament light chain, and P-tau [203]. In long COVID-19, autoimmune responses to myelin basic protein, which are indicative of demyelination, correlate with peripheral manifestations of immunological activation [210]. In Parkinson’s disease, the intensity of depression symptoms is strongly correlated with elevated brain injury markers (NSE and S100B), which are linked to peripheral IRS activation [211].

Altered lipid metabolism may be linked to functional and anatomical alterations in the brain. Elevated BMI and central obesity predict reduced gray matter quantity and atrophy, particularly in the frontal lobe, precuneus, and midbrain [212, 213]. Individuals with greater subcutaneous and visceral belly fat experience a reduction in brain volume during midlife, particularly in areas associated with cognition, memory, and executive processes, such as the temporal lobe [214]. Notably, compared with visceral fat, subcutaneous fat, which accounts for up to 90% of total body fat, is a more accurate predictor of reduced brain volume. The impact of fat mass on brain mass reduction is attributed to the correlation between elevated body fat and heightened proinflammatory status, as well as enhanced oxidative stress, as measured by isoprostanes [215].

The activation of M1 microglia and their effector activities, including phagocytosis and inflammatory signaling, are partially regulated by lipid metabolism, with slight alterations in microglial lipid metabolism linked to neuroinflammation [216]. Mice fed high-fat diets and those with hypercholesterolaemia exhibit activated microglia and astrocytes, which are correlated with compromised working memory [217]. In the periphery, cholesterol uptake induces persistent IRS activation, whereas in the CNS, free cholesterol promotes neuroinflammation [218]. Moreover, neurons with elevated cholesterol buildup exhibit heightened vulnerability to excitotoxicity [219]. Elevated free cholesterol may compromise the integrity of the blood–brain barrier [220] and facilitate IRS activation, potentially leading to enhanced translocation of activated M1 macrophages and Th1 cells into the CNS [220, 221].

### The acute phase of MDD, neurotoxicity, and neuroprotection

**Fig. 4:**
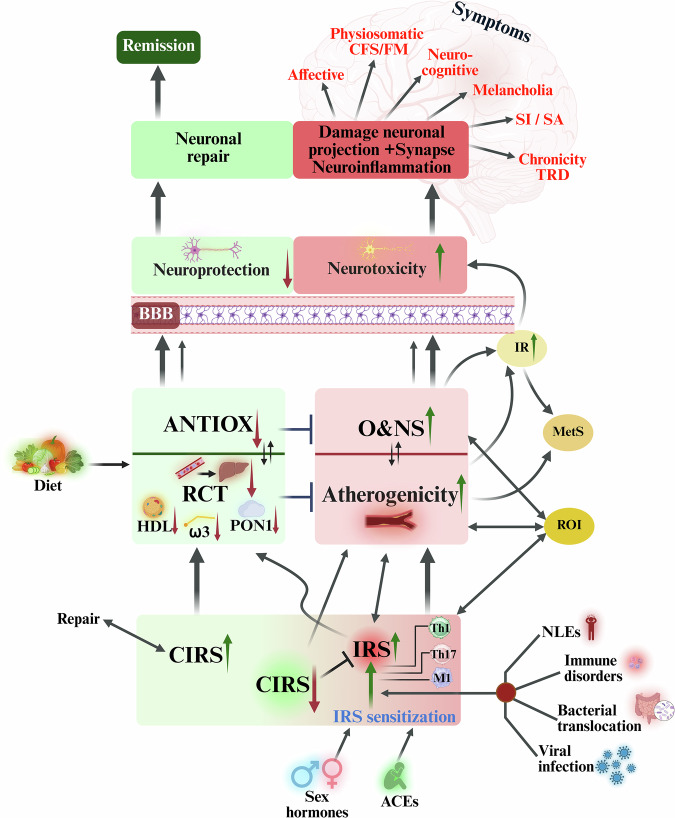
Pathways that cause neurotoxicity in major depression and those that result in diminished neuroprotection. ω3 omega-3 PUFAs, ACEs adverse childhood experiences, ANTIOX antioxidant, BBB blood–brain barrier, CFS chronic fatigue syndrome, CIRS compensatory immunoregulatory system, FM fibromyalgia, HDL high-density lipoprotein cholesterol, IR insulin resistance, IRS immune-inflammatory system, O&NS oxidative and nitrosative stress, PON paraoxonase, M1 M1 classical macrophage, MetS metabolic syndrome, NLE negative life events, RCT reverse cholesterol transport, ROI recurrence of illness, SA suicide attempts, SI suicidal ideation, Th T helper, TRD treatment-resistant depression

As previously discussed, a review from 1999 indicated that immunological mediators play a role in the imbalance between neurotoxicity and neuroprotection [114]. The several pathways examined in this work that enhance neurotoxicity in MDD and those that result in diminished neuroprotection are shown in Fig. 4. Increased neurotoxicity may be attributed to the cumulative effects of IRS activation, encompassing immune-linked neurotoxicity profiles such as classical M1, Th1, and Th17, along with more atypical forms (see below). This phase involves activated T cells, including CD40L + T cells, as well as the activation of NF-κB and NLRP3, the presence of ROS/RNS, free cholesterol, the formation of oxidation-specific epitopes and nitroso-adducts, and autoimmunity directed against these oxidation-specific epitopes and nitroso-adducts. These mechanisms may contribute to the emergence of atherogenicity and MetS. The presence of the latter may exacerbate immune-linked neurotoxicity and O&NS damage [140]. Furthermore, heightened IR may occur, exacerbating the NIMETOX pathway and potentially inducing neurotoxic consequences. These various pathways may result in BBB breakdown, facilitating the ingress of M1 and Th1 cytokines, activated T cells, neurotoxic TRYCATs, free cholesterol, and other entities, which collectively may induce neuroinflammation and activate M1 microglia and A1 astrocytes, ultimately leading to neuronal dysfunction or structural impairments in neurons, including cell bodies, projections, and synapses [1]. Peripheral-to-central signaling may also occur via multiple complementary mechanisms, including cytokine transport systems, neural pathways, and immune–metabolic signaling cascades [1].

ACEs lead to IRS sensitization, which, when confronted with novel immunological stimuli such as increased LPS load, bacterial translocation, viral reactivation, negative life events, and chronic immune injuries (e.g., neurodegenerative diseases and systemic active autoimmune illnesses), results in the overactivation of sensitized IRS networks.

Sex hormones may additionally play a role in IRS sensitization. Sex hormones influence immune function and may heighten IRS activation, thereby enhancing IRS signaling in susceptible individuals [222]. For example, estrogens can augment cytokine synthesis and immune cell activation, whereas androgens may have immunosuppressive effects, resulting in sex-dependent alterations in immune functions [223]. There is also evidence that sex hormones play a role in immune-associated depressive symptoms [224]. For example, the tryptophan catabolite pathway is significantly more active in women than in men, thereby increasing the production of neurotoxic catabolites [225]. Additionally, changes in sex hormones during the menstrual cycle and the prenatal and postnatal periods may further trigger the IRS [222, 226, 227]. Testosterone, on the other hand, may suppress TLR4 cell surface expression [227].

Reduced activity of (neuro)protective mechanisms, such as CIRS, Treg cells, and antioxidant defenses; diminished expression of neurotrophic factors, such as brain-derived neurotrophic factor (BDNF) and nerve growth factor (NGF); and decreased activity of PON1 and LCAT activity and APOA1 and decreased ω3 PUFA levels, ultimately increasing susceptibility to IRS, O&NS, and atherogenic pathways, ultimately leading to neurotoxicity and neuronal damage. Variants of neuronal-associated and IRS-associated genes may increase the risk of neurotoxicity or diminish neuroprotection [1]. The PON1 Q192R gene variation, which influences the activities of its many catabolite sites, is linked to antioxidant defenses and elevated O&NS and MDD [228, 229].

### Metabolomics and lipidomics assays and severe inpatient MDD

Previous metabolomics and lipidomics studies have indicated that MDD, often comorbid with metabolic disease, is characterized by multisystem metabolic disturbances affecting energy metabolism, amino acid pathways, and lipid homeostasis [230–234]. More recent research conducted in China has shown that metabolomic and lipidomic signatures define a cross-validated phenotype of MDD [235, 236]. These signatures accurately predict MDD and strongly predict important phenome features, including overall severity of depression, physiosomatic symptoms, suicidal behaviors, and ROIs. These recent studies were able to elucidate increases in various functional metabolic modules, including diacylglycerol lipotoxicity (diacylglycerol-driven proinflammatory signaling) and ceramide-mediated lipotoxicity, membrane phospholipid remodeling (altered membrane lipid composition), mitochondrial redox dysfunction or oxidative balance, and ether-lipid and plasmalogen depletion (loss of protective antioxidant membrane lipids). Notably, these metabolic changes were not influenced by metabolic syndrome, BMI, sex, or age, suggesting that they are indicative of fundamental pathophysiological processes rather than metabolic comorbidities [235, 236].

Importantly, these data suggest a transition to proinflammatory lipid signaling, which, as described above, is known to facilitate NF-κB activation, oxidative stress, and endothelial dysfunction [235, 236]. Moreover, indicators of plasmalogen depletion add to the deficiencies in antioxidant defenses discussed above. Therefore, a self-sustaining lipid–redox–inflammatory cycle is established, which further drives NIMETOX pathway activation. In summary, these results establish a nonmetabolic syndrome, immune–redox lipid-remodeling phenotype in IMDD that involves the NIMETOX pathway and contributes to the severity, stage, and progression of the disease [235, 236].

**Fig. 5:**
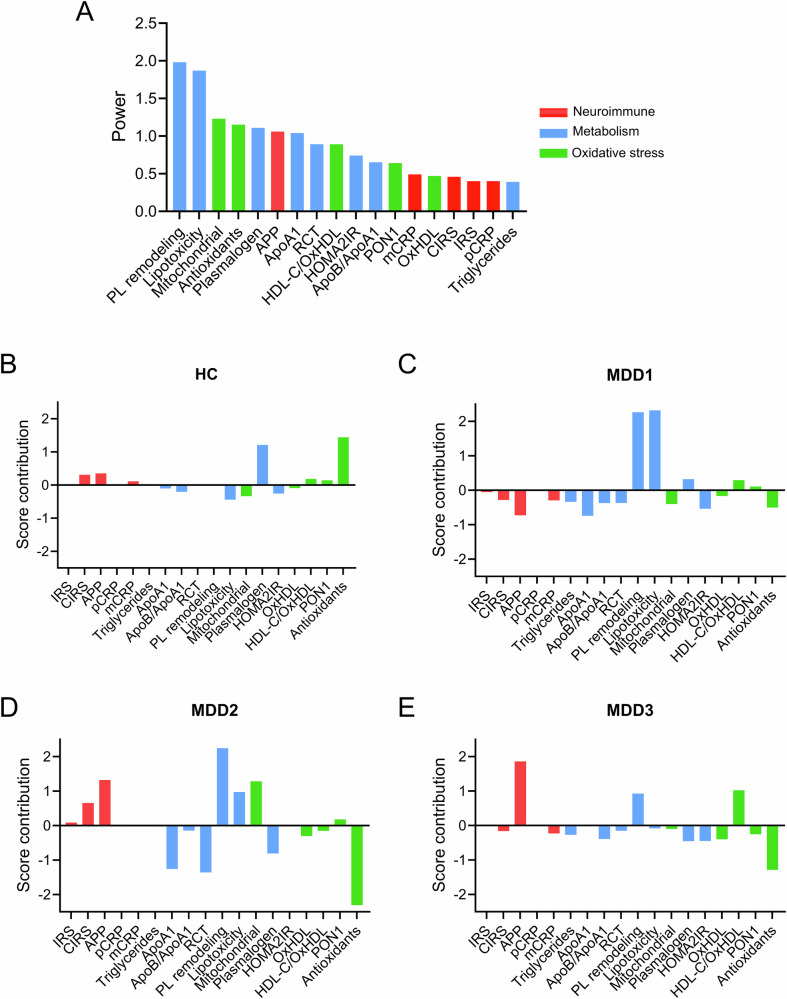
Partial least square discriminant analysis (PLS-DA) with variable importance projection (VIP) scores and PLS-based casewise contribution profiles.** A** The results of partial least squares discriminant analysis (PLS-DA) indicate the variable significance in VIP scores for all biomarkers differentiating patients with MDD from healthy controls. Score contributions for each case derived from the above biomarkers via PLS-DA, illustrating individualized profiles based on relative power in predicting the severity of MDD in one healthy control (**B**) and three MDD patients (**C–E**). PL: phospholipid; APP: acute-phase protein response; RCT: reverse cholesterol transport; HDL-C: high-density lipoprotein cholesterol; OxHDL: oxidized HDL-C; HOMA2IR: homeostatic model assessment 2-insulin resistance; ApoB: apolipoprotein B; ApoA1: apolipoprotein A1; PON1: paraoxonase 1; mCRP: monomeric C-reactive protein; pCRP: pentameric C-reactive protein; IRS: immune-inflammatory response system; CIRS: compensatory immunoregulatory system

To illustrate how nomothetic modeling underpins precision psychiatry [8–10, 144, 237, 238], Fig. 5 presents a cross-validated partial least squares discriminant analysis model integrating neuroimmune (acute phase response, serum monomeric/pentameric CRP, IRS/CIRS), metabolic (serum triglycerides, ApoA1, ApoB/ApoA1 ratio, RCT index, phospholipid remodeling, lipotoxicity, mitochondrial dysfunction, ether lipids/plasmalogens, and an insulin resistance index), and redox (oxHDL, HDL-C/oxHDL ratio, PON1, total antioxidant status) pathways. The model identified three significant latent components explaining 79.0% of the variance, with randomization confirming model validity (Q² = 0.537) and cross-validation indicating high predictive accuracy ( > 90%). Variable importance analysis (Fig. 5A) revealed that single biomarkers (e.g., serum CRP and triglycerides) have limited discriminative power, whereas integrated domains—phospholipid remodeling, lipotoxicity, and mitochondrial dysfunction—together with antioxidant status, acute phase response, and reduced ApoA1—are the principal contributors.

Importantly, the model enables the derivation of individualized contribution scores (Fig. 5B–E), revealing marked heterogeneity in NIMETOX pathway activation. Controls exhibit low activation of detrimental pathways and preserved protective mechanisms (e.g., antioxidant capacity, plasmalogens, CIRS), whereas patients display distinct combinatorial patterns, most frequently driven by phospholipid remodeling and lipotoxicity, with additional contributions from the acute phase inflammatory response and decreased antioxidant defenses in some cases.

These findings demonstrate that nomothetic, data-driven models capture the integrated NIMETOX biology of MDD and can be translated into individualized mechanistic profiles, thereby bridging systems-level NIMETOX dysfunction with precision psychiatry.

## Future research directions

The integration of deep phenotyping and artificial intelligence has resulted in remarkable progress in the diagnosis, drug discovery, and treatment of medical conditions using the precision medicine approach. Likewise, deep phenotyping and machine learning methodologies combined in the nomothetic precision psychiatry approach have significantly enhanced our molecular understanding of the different MDD phenotypes and features and their relationships with NIMETOX pathways [8, 229, 238].

First, this approach assesses the patients’ condition through quantitative clinical and biomarker scores rather than merely considering the presence or absence of MDD as a binary variable (thus, only one bit of information). This methodology transitions the diagnosis of significant psychiatric disorders from a traditional binary framework to a more nuanced clinical evaluation grounded in deep phenotyping. It concurrently enhances the precision of the associations between biomarker composite panels and emerging phenome data [8, 229, 238].

Second, the application of these novel nomothetic models revealed that the recurrence of illness (ROI), which is a composite measure derived from the lifetime number of depressive episodes, suicide attempts, and suicidal ideation, emerged as a significant determinant of the severity of the index MDE and of all NIMETOX pathways, including the gut dysbiosis enterotype [239]. In accordance with our review in “Clinical features or phenotypes of depression”, the NIMETOX pathway is strongly associated with suicidal ideation and attempts and the severity of depression, anxiety, and chronic fatigue [140].

Third, when pattern recognition methods are employed, MDD can be categorized into distinct subgroups, specifically major dysmood disorders and simple dysmood disorders. In this classification, major dysmood disorder (largely overlapping with depression, with melancholia and psychotic features and highly recurrent depression) is defined by a higher ROI and/or pronounced symptoms of depression, anxiety, and physiosomatic manifestations, whereas simple dysmood disorder is characterized by a lower ROI index and a less severe presentation of MDD [10]. Furthermore, we conducted an examination of the NIMETOX profiles pertaining to mild outpatient MDD patients, some of whom are currently experiencing partial remission or mild MDD. Table 4 illustrates that major and simple dysmood disorder and outpatient MDD, in conjunction with an increasing ROI, are linked to distinctly different immunobiological pathways [140].

**Table 4:** Neuroimmune, metabolic, and oxidative stress (NIMETOX) pathways in patients with different major depressive disorder (MDD) phenotypes

Profiles	MDD and ROI	SDMD	OMDD	MDMD
**IRS**	**↑↑**	**↔**	**↔**	**↑↑**
**CIRS**	**↑**	**↓↓**	**↔**	**↑**
**Atherogenicity**	**↑**	Preatherogenic	**↑**	**↑**
**Reverse cholesterol transport**	**↓**	**↓**	**↓**	**↓**
**Insulin resistance**	**↔**	**↔**	**↔**	**↔**
**O&NS**	**↑**	**↑**	**↑**	**↑↑**
**Antioxidant defenses**	**↓**	**↓**	**↓**	**↓↓**

*IRS* immune-inflammatory response system, *CIRS* compensatory immune-regulatory system, *O&NS* oxidative and nitrosative stress, *ROI* recurrence of illness, *SDMD* simple dysmood disorder, *OMDD* outpatients’ major depressive disorder, *MDMD* major dysmood disorder

Consequently, in the contexts of simple dysmood disorder and outpatient MDD, there was a complete absence of evidence supporting the classical inflammatory profiles characterized by M1 and Th1 activation. In contrast, simple dysmood disorder is characterized by a depleted CIRS, which may enable a limited number of neurotoxic cytokines and chemokines to exert immune-linked neurotoxicity effects [240]. In the context of outpatient MDD, an upregulated cytokine and chemokine network was identified using pattern recognition methods, suggesting an increase in immune-linked neurotoxicity, alongside a downregulated network that reflects diminished neuroprotection [241]. All the distinct phenotypes demonstrated elevated levels of O&NS, as well as increased atherogenicity potential, but diminished RCT and antioxidant defenses. Consequently, the prevailing contemporary concept of “inflammatory depression” or “immune-mediated depression” is largely incorrect because most patients with MDD, if not all, demonstrate the presence of one or more NIMETOX pathways, whereas the activation of IRS, M1, Th1, and Th17 cells seems to be restricted to the acute phase of severe IMDD or MDMD, especially in those with increasing ROI. Compared with the (partial) remission phase, the acute phase of MDD may exhibit a markedly distinct NIMETOX profile, with the former characterized by elevated immune-linked neurotoxicity levels and the latter characterized by CIRS activities focused on healing and restoring immune equilibrium.

Fourth, the manifestation of MetS exacerbates immune-related neurotoxicity, and the interaction between increased atherogenicity and IRS biomarkers significantly predicts the severity of the condition [140]. In the context of various depression phenotypes, insulin resistance did not increase in the context of the MDD phenotypes. However, importantly, insulin resistance plays a considerable role in exacerbating the severity of the condition when the aforementioned NIMETOX biomarkers are considered [140].

Future clinical research endeavors should consider the various features that modulate NIMETOX pathways, specifically the ROI and MetS, as well as the various phenotypes of MDD and the phase of the index episode, when this disorder is investigated through the application of advanced machine learning-based composite scores to assess NIMETOX functions. In pursuit of this objective, it is essential to conduct multiplex, transcriptomics and other panomics (genomics, metagenomics, lipidomics, and metabolomics) investigations that provide comprehensive insights into cytokines, chemokines, growth factors, O&NS, atherogenicity profiles, and intracellular networks rather than focusing solely on a few biomarkers, such as CRP, IL-6, TNF-α, and triglycerides. Special emphasis should be placed on the genetic architecture of NIMETOX pathways, including the identification of relevant NIMETOX variants, as well as the epigenetic regulation of these pathways through DNA methylation at NIMETOX-associated differentially methylated regions.

Moreover, the processes involved in the discovery of novel drug targets for MDD/MDE should be grounded in intricate intracellular signaling networks that are fundamental to the NIMETOX pathway. This includes a thorough examination of the imbalances present in neurotoxic versus and neuroprotective paths, such as the dynamics between IRS and CIRS profiles, neurotoxic T effector cells and neuroprotective Treg cell populations, as well as the interplay between atherogenicity and RCT, and O&NS versus antioxidant mechanisms. Future research aimed at identifying the true drug targets in MDD should concentrate on a comprehensive investigation of the molecular NIMETOX pathways in association with immunometabolism, nutritional immunity, and aberrations in the regulation of IRS, microglial and astroglial functions by lipids. Additionally, it is imperative to consider the pathways associated with the innate and adaptive immune systems, the role of autoimmunity in lipid structures and natural IgM autoimmunity in oxidatively specific and nitroso-modified epitopes, nutritional immunity, metabolic endotoxemia, and immunometabolic processes. Furthermore, one must investigate the associations between peripheral NIMETOX pathways and microglial activation A1 astroglial phenotypes via breakdown of the BBB and how these pathways may contribute to heightened neurotoxicity and subsequent neuronal damage within the CNS.

## Supplementary information

Electronic supplementary file (ESF)
