# Supplementary material for: Unifying the hallmarks of major depression through neuroimmune–metabolic–oxidative (NIMETOX) dysregulation: a mechanistic systems framework
Source: Cell Mol Immunol. 2026 Jul 13;23(8):933–57. doi: 10.1038/s41423-026-01448-1 (PMC13424108; doi:10.1038/s41423-026-01448-1)
Supplement: Supplementary file 1 — Electronic supplementary file (ESF) [file 41423_2026_1448_MOESM1_ESM.docx]

**ELECTRONIC SUPPLEMENTARY FILE (ESF)**

**The concept of immune-metabolic depression: the past, present, and emerging research directions.**

Michael Maes, Abbas F. Almulla, Drozdstoj Stoyanov, Yingqiang Zhang*.

**ESF, Table 1a**. Description of the immune profiles used in our studies

| **Immune Profile** | **Members** |
| --- | --- |
| **M1 macrophage** | IL-1β, IL-6, TNF-α, IL-12p70, IL-15, CCL2, CCL5, CXCL1, CXCL8, CXCL9, CXCL10 |
| **M2 macrophage** | IL-10, IL-4, IL-13, VEGF, PDGF, sIL-1RA |
| **z M1 – z M2** | zM1 – zM2: an increase indicates M1 polarization |
| **T helper (Th)-1** | IL-2, sIL-2R, IFN-a, IFN-γ, IL-12p70, IL-16, TNF-α, TNF-β |
| **Th-2** | IL-4, IL-5, IL-9, IL-13, IL-10, IL-6 |
| **Th-1 - Th-2** | zTh-1 – zTh-2: an increase indicates Th-1 polarization |
| **Th-17** | IL-6, IL-17 |
| **Immune-inflammatory response system (IRS)** | IL-1α, IL-1β, IL-6, TNF-α, IL-12p70, IL-15, IL-16, IL-17, IL-18, CCL2, CCL3, CCL4, CCL5, CCL7, CCL11, CXCL1, CXCL8, CXCL9, CXCL10, IL-2, IFN-α, IFN-γ, TNF-α, TNF-β, TRAIL, GM-CSF, M-CSF, G-CSF, SCGF |
| **Compensatory immunoregulatory system (CIRS)** | IL-4, IL-10, sIL-1RA, sIL-2R |
| **z IRS – z CIRS** | Z IRS – z CIRS: an increase indicates breakdown of immune tolerance; a decrease indicates increased immunoregulation or even immunosuppression |
| **Immune-linked neurotoxicity (ILNT)** | IL-1β, IL-6, TNF-α, TRAIL, IL-2, IFN-γ, IL-12p70, IL-16, IL-17, CCL2, CCL3, CCL5, CCL11, CXCL1, CXCL8, CXCL10, GM-CSF, M-CSF |

**ESF, Table 1b**. Cytokines, chemokines, colony-stimulating factors, and growth factors used to compute the immune profiles listed in ESF, Table 1a

| **Protein abbreviations** | **Gene Symbol** | **Protein name / alias** |
| --- | --- | --- |
| **IFN-α2** | **IFNA2** | Interferon-α2 |
| **IFN-γ** | **IFNG** | Interferon-γ |
| **IL-1α** | **IL1A** | Interleukin-1α |
| **IL-1β** | **IL1B** | Interleukin-1β |
| **sIL-1RA** | **IL1RN** | Soluble interleukin-1 receptor antagonist |
| **IL-2** | **IL2** | Interleukin-2 |
| **sIL-2R** | **IL2RA** | Soluble interleukin-2 receptor |
| **IL-4** | **IL4** | Interleukin-4 |
| **IL-5** | **IL5** | Interleukin-5 |
| **IL-6** | **IL6** | Interleukin-6 |
| **IL-9** | **IL9** | Interleukin-9 |
| **IL-10** | **IL10** | Interleukin-10 |
| **IL-12p70** | **IL12RB1** | Interleukin-12 p70 |
| **IL-12p40** | **IL12RB1** | Interleukin-12 p40 |
| **IL-13** | **IL13** | Interleukin-13 |
| **IL-15** | **IL15** | Interleukin-15 |
| **IL-16** | **IL16** | Interleukin-16 |
| **IL-17** | **IL17A** | Interleukin-17 |
| **IL-18** | **IL18** | Interleukin-18 |
| **TNF-α** | **TNF** | Tumor necrosis factor-α |
| **TNF-β** | **LTA** | Tumor necrosis factor-β or lymphotoxin-alpha (LT-α) |
| **TRAIL** | **TNFSF10** | TNF-related apoptosis-inducing ligand (TRAIL) or tumor necrosis factor ligand superfamily member 10 (TNFSF10) |
| **MIF** | **MIF** | Macrophage migration inhibitory factor-like protein (MIF) or glycosylation-inhibiting factor |
| **G-CSF** | **CSF3** | Granulocyte colony stimulating factor (G-CSF) or colony stimulating factor 3 (CSF3) |
| **M-CSF** | **CSF1** | Macrophage colony-stimulating factor (M-CSF) or colony stimulating factor 1 (CSF1) |
| **GM-CSF** | **CSF2** | Granulocyte-macrophage colony-stimulating factor (GM-CSF) or colony-stimulating factor 2 (CSF2) |
| **CCL2 or MCP1** | **CCL2** | C-C motif chemokine ligand 2 (CCL2) or monocyte chemoattractant protein 1 (MCP1) |
| **CCL3 or MIP-1α** | **CCL3** | C-C motif Chemokine ligand 3 (CCL3) or macrophage inflammatory protein 1-alpha (MIP-1α) |
| **CCL4 or MIP-1β** | **CCL4** | C-C motif chemokine ligand 4 (CCL4) or macrophage inflammatory protein 1β (MIP-1β) or lymphocyte activation gene 1 protein |
| **CCL5 or RANTES** | **CCL5** | C-C motif chemokine ligand 5 (CCL5) or regulated upon activation, normally T-expressed, and presumably Secreted (RANTES) |
| **CCL7 or MCP3** | **CCL7** | C-C motif chemokine ligand 7 (CCL7) or monocyte-chemotactic protein 3 (MCP3). |
| **CCL11 or Eotaxin** | **CCL11** | C-C motif chemokine ligand 11 (CCL11) or eosinophil chemotactic protein |
| **CCL27 or CTACK** | **CCL27** | C-C motif chemokine ligand 27 (CCL27) or cutaneous T-cell attracting chemokine (CTACK) |
| **CXCL1 or GRO-α** | **CXCL1** | C-X-C motif chemokine 1 (CXCL1) or growth-regulated alpha protein (GRO) |
| **CXCL8 or IL-8** | **CXCL8** | C-X-C motif chemokine ligand 8 (CXCL8) or interleukin-8 (IL-8) |
| **CXCL9 or MIG** | **CXCL9** | C-X-C motif chemokine ligand 9 (CXCL9) or monokine induced by gamma interferon (MIG) |
| **CXCL10 or IP10** | **CXCL10** | C-X-C motif chemokine ligand 10 (CXCL10) or Interferon gamma-induced protein 10 (IP10) |
| **PDGF** | **PDGFA** | Platelet derived growth factor (PDGF) |
| **SCGF-β or CLEC11A** | **CLEC11A** | Stem cell growth factor (SCGF) or C-type lectin domain family 11 member A (CLEC11A) |
| **VEGF** | **VEGFA** | Vascular endothelial growth factor (VEGF) |
